# Molecular Genetic Contributions to Social Deprivation and Household Income in UK Biobank

**DOI:** 10.1016/j.cub.2016.09.035

**Published:** 2016-11-21

**Authors:** W. David Hill, Saskia P. Hagenaars, Riccardo E. Marioni, Sarah E. Harris, David C.M. Liewald, Gail Davies, Aysu Okbay, Andrew M. McIntosh, Catharine R. Gale, Ian J. Deary

**Affiliations:** 1 Centre for Cognitive Ageing and Cognitive Epidemiology, University of Edinburgh, 7 George Square, Edinburgh EH8 9JZ, UK; 2 Department of Psychology, University of Edinburgh, 7 George Square, Edinburgh EH8 9JZ, UK; 3 Division of Psychiatry, University of Edinburgh, Morningside Terrace, Edinburgh EH10 5HF, UK; 4 Medical Genetics Section, University of Edinburgh Centre for Genomics and Experimental Medicine and MRC Institute of Genetics and Molecular Medicine, Western General Hospital, Crewe Road, Edinburgh EH4 2XU, UK; 5 Queensland Brain Institute, The University of Queensland, St Lucia, Queensland 4072, Australia; 6 Department of Applied Economics, Erasmus School of Economics, Erasmus University Rotterdam, Burgemeester Oudlaan 50, Rotterdam 3062 PA, the Netherlands; 7 Department of Epidemiology, Erasmus Medical Center, Gravendijkwal 230, Rotterdam 3015 GE, the Netherlands; 8 Erasmus University Rotterdam Institute for Behavior and Biology, Gravendijkwal 230, Rotterdam 3062 PA, the Netherlands; 9 MRC Lifecourse Epidemiology Unit, University of Southampton, Tremona Rd, Southampton SO16 6YD, UK

**Keywords:** GWAS, genetics, genetic correlation, UK Biobank, socioeconomic status, SES, income, social deprivation

## Abstract

Individuals with lower socio-economic status (SES) are at increased risk of physical and mental illnesses and tend to die at an earlier age [1, 2, 3]. Explanations for the association between SES and health typically focus on factors that are environmental in origin [4]. However, common SNPs have been found collectively to explain around 18% of the phenotypic variance of an area-based social deprivation measure of SES [5]. Molecular genetic studies have also shown that common physical and psychiatric diseases are partly heritable [6]. It is possible that phenotypic associations between SES and health arise partly due to a shared genetic etiology. We conducted a genome-wide association study (GWAS) on social deprivation and on household income using 112,151 participants of UK Biobank. We find that common SNPs explain 21% of the variation in social deprivation and 11% of household income. Two independent loci attained genome-wide significance for household income, with the most significant SNP in each of these loci being rs187848990 on chromosome 2 and rs8100891 on chromosome 19. Genes in the regions of these SNPs have been associated with intellectual disabilities, schizophrenia, and synaptic plasticity. Extensive genetic correlations were found between both measures of SES and illnesses, anthropometric variables, psychiatric disorders, and cognitive ability. These findings suggest that some SNPs associated with SES are involved in the brain and central nervous system. The genetic associations with SES obviously do not reflect direct causal effects and are probably mediated via other partly heritable variables, including cognitive ability, personality, and health.

## Highlights

• Common SNPs explain 21% of social deprivation and 11% of household income
• Two loci attained genome-wide significance for household income
• Genes in these loci have been linked to synaptic plasticity
• Genetic correlations were found between both measures of SES and many other traits

## Teaser

Individuals with lower socio-economic status (SES) are at increased risk of physical and mental illnesses. Hill et al. find extensive genetic correlations between SES and health, psychiatric, and cognitive traits. This suggests that the link between SES and health is driven, in part, by a shared genetic association.

## Results and Discussion

**Figure 1:**
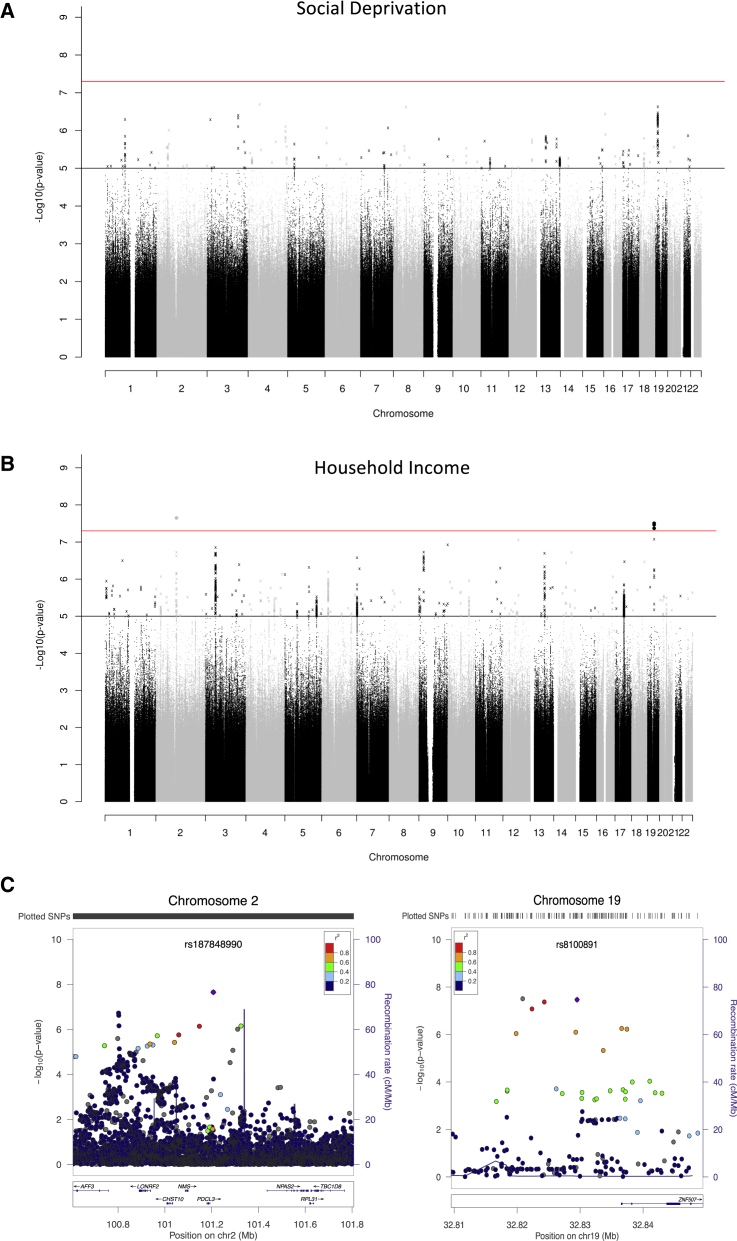
Results of Genome-wide Analysis on Social Deprivation and Household Income (A) Manhattan plot of –log_10_ (p values) for social deprivation. The red line indicates genome-wide significance (p < 5 × 10^−8^). The black line indicates values that were suggestive of statistical significance (p < 1 × 10^−5^). See also Figure S1. (B) Manhattan plot of –log_10_ (p values) for household income. The red line indicates genome-wide significance (p < 5 × 10^−8^). The black line indicates values that were suggestive of statistical significance (p < 1 × 10^−5^). See also Figure S1. (C) Regional association plots for household income of SNPs that attained genome-wide significance (p < 5 × 10^−8^). rs187848990 is on the left; rs8100891 is on the right. The most significant SNP in these regions is represented with a purple diamond. Each circle represents an individual SNP, and the color indicates pairwise linkage disequilibrium with the most significant SNP in the region (as calculated from 1000 Genomes in November 2014). The solid blue line indicates the recombination rate, and the –log_10_ p values are shown on the y axis. See also Table S1 and Figures S1–S4.

Using GCTA-GREML [7], we first estimated the heritability of each of the SES variables in the UK Biobank sample. A total of 21% (SE = 0.5%) of phenotypic variation in social deprivation, as measured using Townsend scores, and 11% (SE = 0.7%) of household income was explained by the additive effects of common SNPs. Next, genome-wide association analyses for social deprivation and household income were performed using an imputed dataset that combined the UK10K haplotype and 1000 Genomes Phase 3 reference panels; details can be found at http://biobank.ctsu.ox.ac.uk/crystal/refer.cgi?id=157020. We found no genome-wide significant findings associated with social deprivation (see Figure 1A and Figure S1). For household income, four SNPs attained genome-wide significance (p < 5 × 10^−8^): rs187848990 on chromosome 2, and rs7252896, rs7255223, and rs8100891 on chromosome 19 (see Figure 1B, Figure S1, and Table S1).

The “clump” function in PLINK [8] was used to identify patterns of linkage disequilibrium in the dataset and showed that these four SNPs were located in two independent regions (Figure 1C). The region on chromosome 2 spanned 583 kb, and the most significant SNP was rs187848990 (p = 2.325 × 10^−8^). This region contains five genes: *AFF3*, *CHST10*, *LONRF2*, *NMS*, and *PDCL3*. The *AFF3* gene has previously been associated with intellectual disability [9], and *CHST10* is involved with synaptic plasticity [10]. The region on chromosome 19 spanned 18 kb, with the most significant SNP being rs8100891, p = 3.423 × 10^−8^. This region contains the gene *ZNF507*, which has been implicated in neurodevelopmental disorders including schizophrenia [11], a disorder that affects cognitive function and that shows a strong genetic correlation with intelligence [12]. It is possible, therefore, that these genetic associations with SES may be mediated, in part, through cognitive ability; it is well established that an individual’s level of cognitive ability is correlated with their SES [13].

Using the GTEx database (http://www.broadinstitute.org/gtex/), cis-eQTL associations were identified for the four household income genome-wide significant SNPs (Table S1). For this study, data mining of regulatory elements was restricted to normal tissues. There was evidence of regulatory elements associated with all four of the genome-wide significant SNPs (Table S1).

We next sought to replicate the four genome-wide significant SNPs in a sample of ∼200,000 individuals who were assessed on the number of years of schooling completed, as this variable is often used as a measure of SES. Summary statistics were made available from the Social Science Genetic Association Consortium’s GWAS of educational attainment [14], with data from UK Biobank, UK-based cohorts, and 23andMe being omitted from the analysis. Three of our genome-wide significant SNPs were successfully replicated using years of education as the phenotype, rs187848990 on chromosome 2 (β = 0.066, p = 0.047), and rs7255223 (β = 0.044, p = 7.28 × 10^−4^) and rs8100891 (β = 0.044, p = 7.62 × 10^−4^) on chromosome 19. rs7252896 was not included in the education data and thus could not be replicated. We then sought to use a SNP that was in high linkage disequilibrium (LD) with rs7252896 to use as a proxy SNP for replication; however, there were no SNPs in the education dataset that were in LD with rs7252896 (r^2^ of greater than 0.5), excluding rs7255223 and rs8100891.

We also used this education summary GWAS dataset to derive genetic correlations between both the social deprivation and household income variables in UK Biobank. A genetic correlation of 0.548 (SE = 0.054, p = 1.796 × 10^−24^) was found between social deprivation and years of education, and there was a genetic correlation of 0.903 (SE = 0.040, p = 4.135 × 10^−115^) between income and years of education. These substantial genetic correlations indicate that the two measures of SES, as measured in UK Biobank, have a very similar genetic architecture with a third SES variable—education—measured in an independent sample.

Next, we used polygenic profile scores, derived using the social deprivation and household income variables’ GWASs in UK Biobank, to predict social deprivation (using the Scottish Index of Multiple Deprivation, SIMD) and household income in an independent sample, Generation Scotland: Scottish Family Health Study (GS:SFHS) [15, 16]. Polygenic profile scores, calculated using marker weights from the social deprivation GWAS in UK Biobank, produced highly significant associations at each p value threshold with SIMD in GS:SFHS, with the most predictive score being that which was derived using all SNPs (β = 0.079, SE = 0.008, r^2^ = 0.008, p = 2.26 × 10^−5^). Similarly, a polygenic score derived using household income in UK Biobank predicted a significant proportion of phenotypic variance for household income in GS:SFHS at each of the p value thresholds used, with polygenic scores derived using a p value threshold of 0.5 being the most predictive (β = 0.052, SE = 0.008, r^2^ = 0.003, p = 5.07 × 10^−11^). The results of the polygenic profile scores illustrate that the molecular genetic architecture of these SES variables, as measured in the UK Biobank datasets, overlaps with that of GS:SFHS, indicating that the same genetic variants are associated with phenotypic variation in SES in each of these samples. The betas found using the polygenic profile score method predict only a small proportion of the phenotypic variance, which is in line with other phenotypes [17].

Gene-based association testing for the two SES variables in the UK Biobank sample was conducted using MAGMA [18]. Following Bonferroni correction for multiple testing (α = 2.768 × 10^−6^), gene-based association tests identified one gene associated with social deprivation: *ACCSL* on chromosome 11 (p = 3.48 × 10^−7^). For household income, 12 genes showed a significant association: *KANSL1* (p = 8.20 × 10^−8^), *MST1* (p = 1.10 × 10^−7^), *RNF123* (p = 1.19 × 10^−7^), *MAPT* (p = 1.23 × 10^−7^), *APEH* (p = 2.64 × 10^−7^), *BSN* (p = 1.03 × 10^−6^), *PLEKHM1* (p = 1.16 × 10^−6^), *SGCD* (p = 1.30 × 10^−6^), *DAG1* (p = 1.55 × 10^−6^), *CRHR1* (p = 2.39 × 10^−6^), *AMT* (p = 2.39 × 10^−6^), and *ZDHHC11* (p = 2.54 × 10^−6^) (Supplemental Experimental Procedures). The *MAPT*, *KANSL1*, *PLEKHM1*, and *CRHR1* genes have been associated with Alzheimer’s disease [19].

Partitioned heritability analysis was then conducted on both SES phenotypes in UK Biobank [20]. The goal of the partitioned heritability analysis was to determine whether SNPs that are grouped together, according to a specific biological function or role, make an enriched contribution to the total proportion of heritability for each of the SES variables. The functional categories used here overlap considerably, meaning that the heritability measured by all groups, when they are summed, can exceed 100%. By deriving a heritability estimate for functional classes of SNPs across the genome, a significant enrichment was found for conserved regions of the genome. These conserved regions accounted for 2.6% of the SNPs in both SES phenotypes and accounted for 44% (SE = 12%) of the heritability of social deprivation and 53% (SE = 12%) of the heritability for household income. The trend for enrichment in heritability emanating from such regions is consistent with the results from other quantitative traits and diseases [20] and, as such, further highlights the importance of these genetic regions as sources of phenotypic variance across these complex traits. Under models of neutral selective pressure, these regions accumulate base-pair substitutions at a lower rate than other regions of the genome, indicative of their being regions where mutation results in the production of phenotypic variance susceptible to the effects of purifying selection. Genetic variance within these regions may highlight a role for disease-causing loci, which in turn might account for some phenotypic variance in SES. However, it is also possible that, as intelligence is phenotypically and genetically associated with many health traits [21] and is thought to be evolutionarily selected for [22], these regions may show their association with SES partly through cognitive differences. These two explanations are not mutually exclusive because, after intelligence is included as a covariate, the associations between adult SES and health outcomes, although attenuated, remain significant [1].

**Figure 2:**
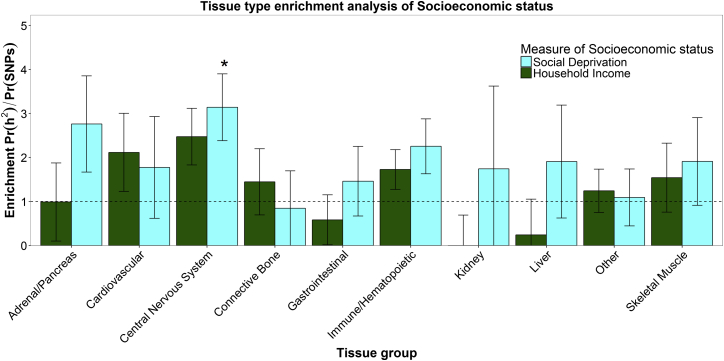
Enrichment Analysis for Social Deprivation and Household Income using the Ten Tissue-Specific Functional Categories The enrichment statistic is the proportion of heritability found in each functional group divided by the proportion of SNPs in each group: Pr(h^2^)/Pr(SNPs). Error bars are jackknife standard errors around the estimate of enrichment. The dashed line indicates no enrichment found when Pr(h^2^)/Pr(SNPs) = 1. Social deprivation (blue) in only one category was significant, as indicated by an asterisk. No significant enrichment was found for any of the categories considered for the household income (green) phenotype.

Partitioned heritability analysis was also used to conduct a cell-specific analysis of ten broad tissue types (see Supplemental Experimental Procedures). Figure 2 shows the results of cell-specific enrichment for social deprivation and household income. For social deprivation, significant enrichment was found in variants exerting an effect within the central nervous system. Variants expressed in the central nervous system accounted for 15% of the total number of SNPs but accounted for 47% (SE = 11%) of the heritability of social deprivation and 37% (SE = 9%) of the heritability of household income. For household income, this did not survive multiple-testing correction.

We next derived genetic correlations, using linkage disequilibrium score (LDS) regression [23], between both measures of SES and a set of 32 phenotypes that have all been shown in some studies to be phenotypically associated with SES. Table S2 provides references describing examples of the phenotypic associations between measures of SES and broadly conceived health variables. Full details of the GWAS that provided summary statistics for each of the 32 phenotypes, along with links to the data, are also provided in Table S2. The direction of effect for the genetic correlations and polygenic profile scores examining Townsend scores was reversed to facilitate a comparison with the household income variable.

**Figure 3:**
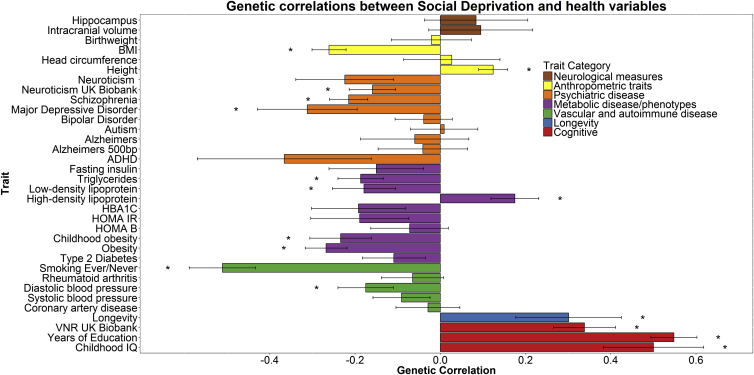
Genetic Correlations between Social Deprivation and Health and Anthropometric Variables The x axis depicts the magnitude of the genetic correlations; the y axis shows each trait. Statistical significance is indicated by an asterisk. FDR correction indicated statistical significance at p = 0.015. Error bars represent standard error using a ratio block jackknife. HOMA B, homeostatic model assessment β cells; HOMA IR, homeostatic model assessment insulin resistance; HbA1c, glycated hemoglobin; ADHD, attention deficit hyperactivity disorder; MDD, major depressive disorder; BMI, body mass index; ICV, intracranial volume. Social deprivation scores were reversed so that a higher Townsend score indicates a higher SES. See also Tables S2, S3, and S4.

**Figure 4:**
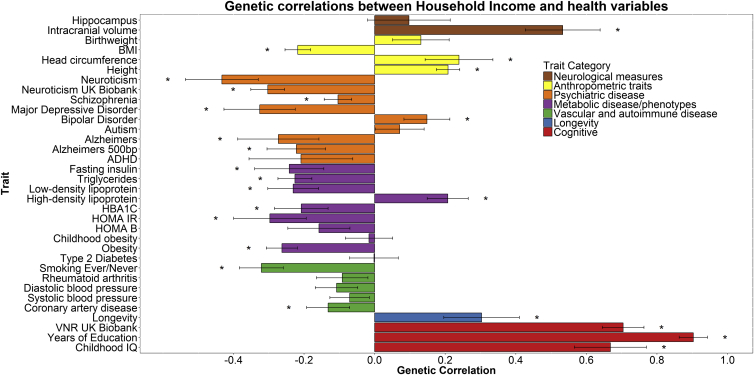
Genetic Correlations between Household Income and Health and Anthropometric Variables The x axis depicts the magnitude of the genetic correlations; the y axis shows each trait. Statistical significance is indicated by an asterisk. FDR correction indicated statistical significance at p = 0.032. Error bars represent standard error using a ratio block jackknife. HOMA B, homeostatic model assessment β cells; HOMA IR, homeostatic model assessment insulin resistance; HbA1c, glycated hemoglobin; ADHD, attention deficit hyperactivity disorder; MDD, major depressive disorder; BMI, body mass index; ICV, intracranial volume. For household income, higher scores represent higher income. See also Tables S2, S3, and S4.

Following false discovery rate (FDR) correction for multiple comparisons, 16 of the 34 genetic correlations were statistically significant for the Townsend social deprivation measure (see Figure 3 and Tables S3), and 24 of the 34 were significant for household income (Figure 4 and Table S3). The large number of genetic correlations found indicates that the molecular genetic associations with SES overlap with many other health-relevant phenotypes. A large degree of overlap was found for variables that are cognitive in nature. Significant genetic correlations were observed, for example, between both measures of SES and childhood cognitive ability (social deprivation, *r*_g_ = 0.500; income, *r*_g_ = 0.667), with participants’ verbal-numerical reasoning scores in the UK Biobank clinic visit (social deprivation, *r*_g_ = 0.338; income, *r*_g_ = 0.711), and also with longevity (social deprivation, *r*_g_ = 0.301; household income, *r*_g_ = 0.303). The direction of effect in each instance indicates that more affluent SES is associated with longer life and higher intelligence. The average age of the participants in the GWAS for childhood intelligence was 11 years, whereas the measurements of SES from UK Biobank were taken at a mean age of 57 years. The finding of a genetic correlation between these two traits may indicate that a set of genetic variants contributes to higher intelligence, which in turn contributes to a higher SES in mid-life. Significant genetic correlations were found between household income and intracranial volume and infant head circumference (*r*_g_ = 0.533 and *r*_g_ = 0.239, respectively).

A noteworthy feature of our findings is that the pattern of genetic correlations between our two measures of SES—one area-based and one individual-based—was very similar, and the genetic correlation between the two measures of SES was high, at 0.871 (SE = 0.064). The Townsend social deprivation score is widely used as a proxy indicator of adult socioeconomic status, usually in the absence of an individual measure. It has been shown to be predictive of cancer incidence, all-cause mortality, and other health outcomes [24]. Such area-level effects may comprise both compositional effects, i.e., effects that can be explained in terms of the characteristics of the residents of those areas, and contextual effects, i.e., effects that can be explained in terms of the characteristics of the areas. Although ecological correlations cannot be used to make causal inferences about individuals—the ecological fallacy—it has been suggested that they arise largely from associations at the individual level [25]. One study found that area-based Townsend scores correlate highly with a similar measure of deprivation calculated at the individual level [26]. In our UK Biobank sample, where the individual-level measure of SES was based on household income alone, its correlation with Townsend score was small to moderate in size (r = 0.24); despite this, the pattern of genetic correlations between these two measures was very similar.

There are at least two explanations for the genetic SES-health correlations found using the LDS regression method. The first is that the genetic correlations might have been found as a result of the same genetic variants being directly involved in two phenotypes. The second is the notion of mediated pleiotropy, which describes situations in which a phenotype is causally related to another, perhaps via other variables; therefore, if a genetic variant is associated with the first phenotype, it will be indirectly associated with the second [27]. Should multiple variants be used to establish pleiotropy, such as when using the LDS regression method, both of these forms of pleiotropy may apply, at different loci. However, because SES has no clear biological analog—it describes the environment of an individual or their status within it—mediated pleiotropy thorough intelligence or personality traits such as conscientiousness, for example, would appear to be a much more likely interpretation than biological pleiotropy; that is, we do not conceive of there being genetic variants directly related to SES measures.

The genetic correlations derived in the current paper cannot distinguish the direction of the effect of this shared genetic association between SES and health and cognitive variables. Whereas it is possible that a greater level of cognitive ability will facilitate an individual’s ability to move to a higher SES, it could also be the case that those in higher SES environments are exposed to environmental stimuli that facilitate their intellectual development. As both SES and cognitive ability are partly heritable, each of these possibilities would result in a genetic correlation between SES and cognitive ability. Mendelian randomization (MR) is a technique that sits above genetic correlations and polygenic profile scores in the so-called hierarchy of evidence [28] for ascertaining causal inference with regard to how genetic contributions act on exposure and outcomes, as well as for testing the direction of association. However, the data required to perform these analyses for all traits are not currently available.

The associations between rs187848990 and rs8100891 with household income, along with the heritability estimates for both measures of SES, are, we think, the result of mediation through other phenotypes. Because genetic differences will not directly result in differences in SES, they may be contributing to differences in variables such as intelligence, personality, resistance to diseases, and other factors, which, in turn, can contribute to differences in SES.

The effect sizes found for each individual SNP were small; however, as has been found for other polygenic traits, it is the combined effect of multiple SNPs that contributes to some of the observable phenotypic variance. Polygenic profile scores were created for 28 health-related phenotypes using published GWAS in all participants with genome-wide SNP data. When predicting into social deprivation based on the Townsend score, polygenic profile scores derived from the summary statistics of 11 GWASs of health-related traits predicted a significant proportion of phenotypic variance (Table S4). When predicting into household income, 26 out of 28 demonstrated statistical significance (Table S4). Polygenic profile scores explained only a very small proportion of variance, but they illustrate that genetic risk for a range of diseases and cognitive ability can predict variance in these two SES measures.

As the income variable used in our GWAS pertained to household income, it may have included multiple individuals from the same household. This may have led to individuals providing a phenotype score that did not reflect their own income, but rather the income of those that they lived with. We sought to determine the number of individuals who co-habited in the UK Biobank data and the degree to which this may have influenced the results of the household income GWAS. We omitted one individual per household, retaining the male where possible (Figure S2), which resulted in a reduced sample size of 88,183 individuals who had provided data on their level of household income. Next, we repeated the GWAS, GREML, gene-based analysis, genetic correlations, and the polygenic profile scores. Using this reduced sample in the household income dataset, two additional suggestive peaks were found on chromosome 9, with the most significant SNPs in each of these regions being rs139128645 (β = −0.22, p = 1.39 × 10^−8^) and rs7467480 (β = 0.027, p = 2.22 × 10^−8^) (Figure S3). The results of the GWAS and the additional analyses were consistent with the likelihood that, by modifying the sample size, there will be minor changes to the test statistics; the results of the GWAS on the full sample for household income and the reduced sample for household income were highly similar. The results did not suggest that a bias had been introduced by there being multiple individuals from a single household. The full results of the reduced-sample GWAS, along with the follow-up analyses, are available from the authors.

The results here show that 21% of people’s differences in area-level social deprivation and 11% of household income can be explained by additive common genetic factors. Four genome-wide significant SNPs were found for household income, leading to the identification of two independent genomic regions containing genes with known associations with intellectual disabilities, synaptic plasticity, and schizophrenia. Extensive genetic correlations were found between both measures of SES and health-related traits, indicating a highly diffuse genetic architecture. These genetic correlations might provide a partial explanation for the phenotypic association between SES and health—the majority of which, we think, is due to environmental factors.

## Experimental Procedures

We examined two measures of SES that were available in UK Biobank (http://www.ukbiobank.ac.uk) [29]. The first measure was the Townsend Social Deprivation Index [30]—a measure of the level of social deprivation in which the participant lives—and the second measure was household income. A total of 112,005 individuals had a Townsend score, of whom 52.53% were female (mean age = 56.91 years, SD = 7.93, range 40–73). A total of 96,900 participants had data pertaining to household income, of whom 50.64% were female (mean age = 56.53 years, SD = 7.95, range 40–73). Participants had undergone genome-wide SNP genotyping; the full details of this can be found in the Supplemental Experimental Procedures.

We used data from the UK Census of 2001 (https://census.ukdataservice.ac.uk/media/215850/Townsend2001.csv) and compared the distribution of Townsend scores from all of England and Wales to those in UK Biobank that had been genotyped. The data from the UK census exclude wards of less than 100 households, which only altered wards in the City of London and the Scilly Isles. The score was first reversed so that a greater Townsend score corresponds to a higher SES. As can be seen in Figure S4, the distribution of the Townsend score from the UK Biobank dataset follows the same trend as that found across England and Wales. This indicates that, whereas those from very low SES environments—corresponding to a Townsend score of less than −10—did not participate in UK Biobank, the distribution of scores is highly similar to what was found across England and Wales (UK Biobank median = 2.3, census of 2001, median = 1.1). The distribution of the income scores can be found in Figure S4.

We conducted separate analyses for the Townsend deprivation score and household income. All phenotypes were adjusted for age, gender, assessment center, genotyping batch, genotyping array, and ten principal components in order to correct for population stratification prior to all analyses. See Supplemental Experimental Procedures for full description of genotyping, imputation, and the phenotypes used.

## Author Contributions

Conceptualization, W.D.H., C.R.G., I.J.D.; Software, D.C.M.L., G.D.; Formal Analysis, W.D.H., S.P.H., R.E.M., G.D.; Data Curation, W.D.H., S.P.H., S.E.H.; Writing – Original Draft, W.D.H., as discussed with I.J.D.; Writing – Review and Editing, W.D.H., S.P.H., R.E.M., S.E.H., D.C.M.L., G.D., A.O., A.M.M., C.R.G., I.J.D.

## Supplemental Information

Document S1. Supplemental Experimental Procedures, Figures S1–S4, and Tables S1 and S3

Document S3. Table S4

Document S4. Article plus Supplemental Information
