# Supplementary material for: Molecular Genetic Contributions to Social Deprivation and Household Income in UK Biobank
Source: Curr Biol. 2016 Nov 21;26(22):3083–9. doi: 10.1016/j.cub.2016.09.035 (PMC5130721; doi:10.1016/j.cub.2016.09.035)
Supplement: Document S1. Supplemental Experimental Procedures, Figures S1–S4, and Tables S1 and S3 [file mmc1.pdf]

Current Biology, Volume *26*

Supplemental Information

Molecular Genetic Contributions to Social Deprivation and Household Income in UK Biobank

W. David Hill, Saskia P. Hagenaars, Riccardo E. Marioni, Sarah E. Harris, David C.M. Liewald, Gail Davies, Aysu Okbay, Andrew M. McIntosh, Catharine R. Gale, and Ian J. Deary

Supplemental Figures and Tables

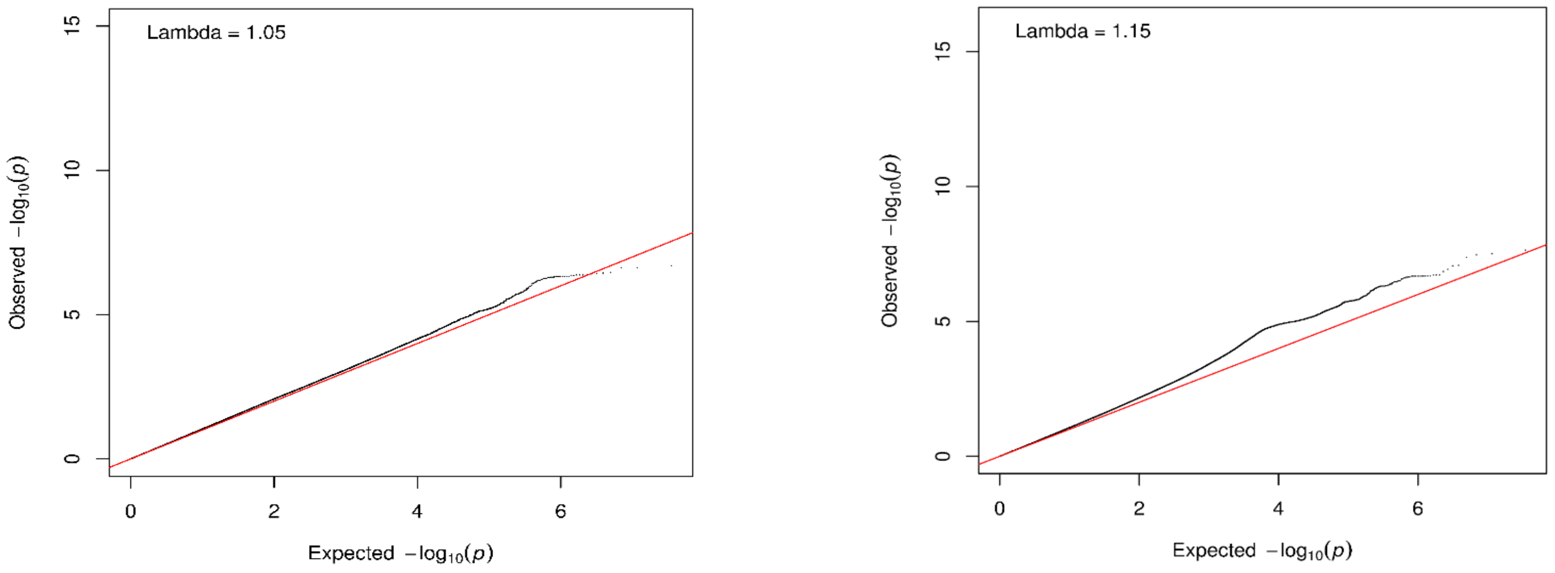

Figure S1. Related to Figure 1. Q-Q plot of social deprivation (left panel) shows that the distribution of $-\log_{10}$ *P*-values follows that which would be expected under the null hypothesis. Q-Q plot of household income (right panel) shows that the distribution of $-\log_{10}$ *P*-values indicates more low *P*-values than would be expected under the null hypothesis.

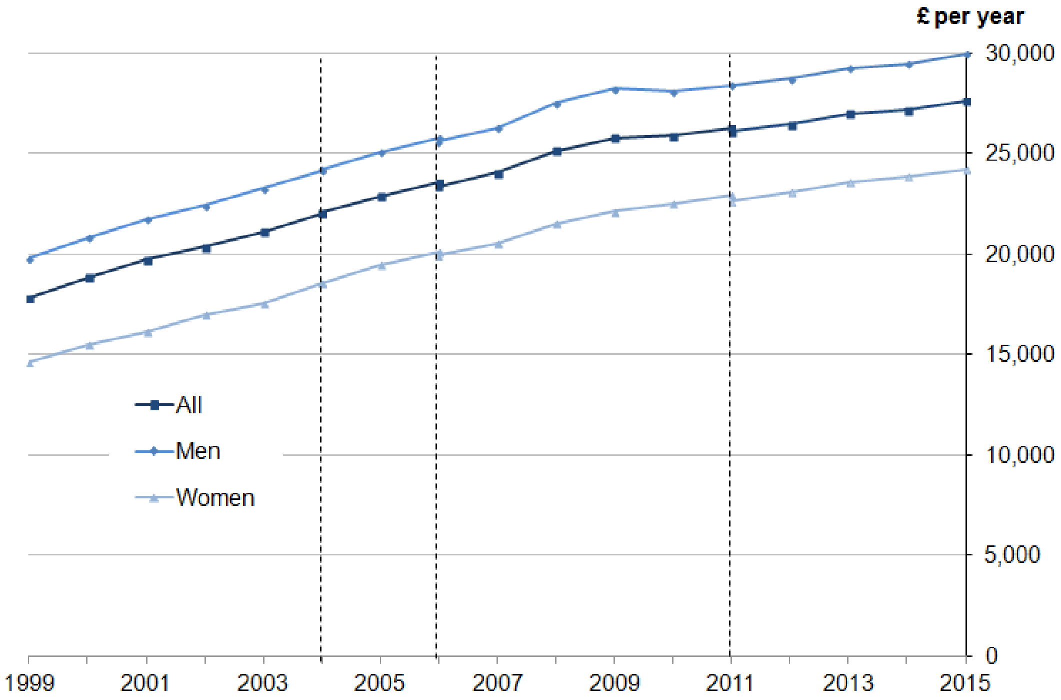

Figure S2. Related to Figure 1. Median full-time gross annual earnings by sex in the UK from 1999 to 2015.

Employees employed for greater than one year and were working full timed defined as greater than 30 hours per week, or 25 for teaching professions. Dashed line indicates discontinuities in the estimates of the Annual Survey of Hours and Earnings.

Figure taken from the Office for National Statistics (http://www.ons.gov.uk/employmentandlabourmarket/peopleinwork/earningsandworkinghours/bulletins/annualsurveyofhoursandearnings/2015provisionalresults#gender-pay-differences).

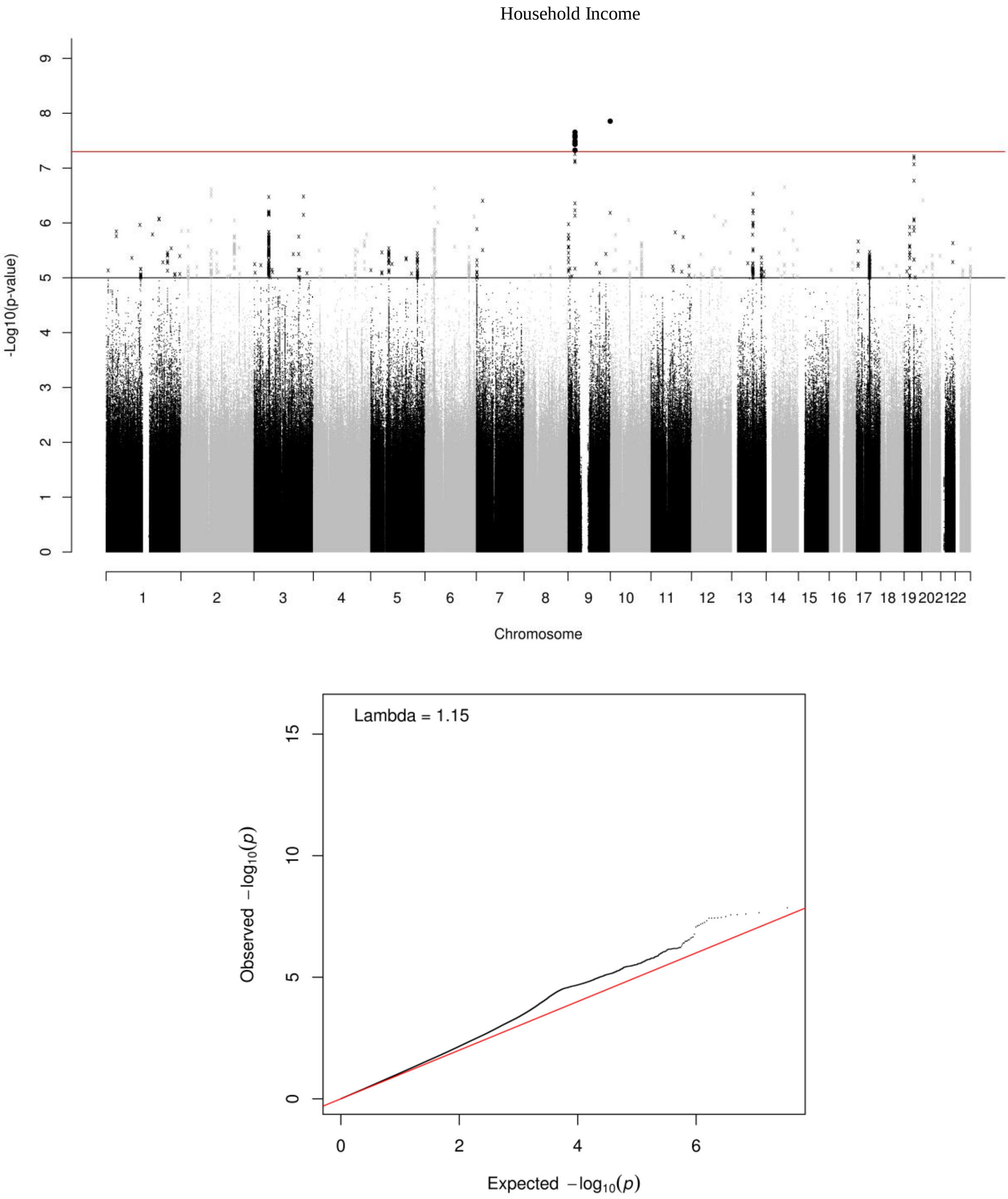

Figure S3. Related to Figure 1. Manhattan and Q-Q plot of the reduced sample size of 88,183. The upper panel shows the Manhattan plot for household income using. The red line indicates genome wide significance ($P < 5 \times 10^{-8}$). The black line indicates values that were suggestive of statistical significance ($P < 1 \times 10^{-5}$). The lower panel shows the Q-Q plot and shows that the distribution of $-\log_{10} P$-values indicates more low P-values than would be expected under the null hypothesis.

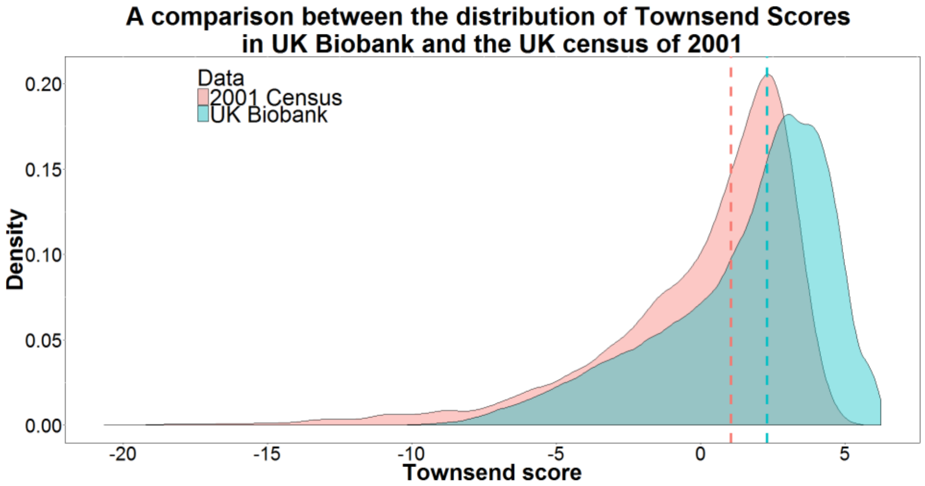

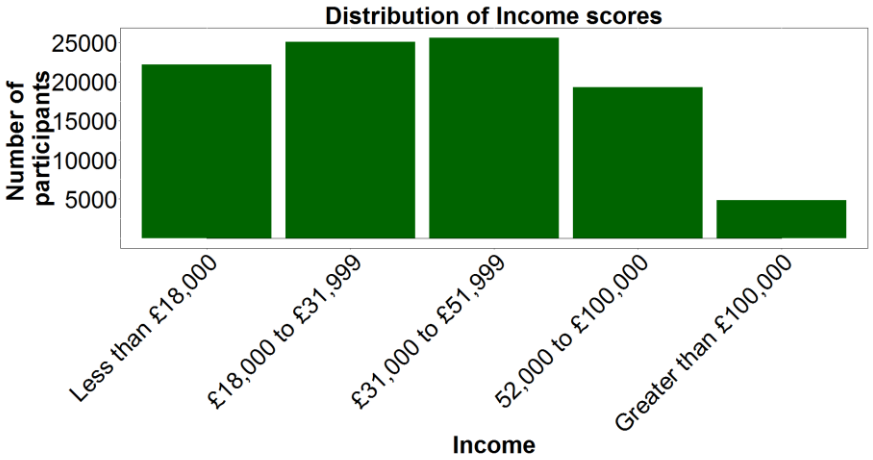

Figure S4. Related to Figure 1. The panel on the left shows the distribution of scores from the Townsend scores in UK Biobank with those taken from the national census in 2001 in England and Wales. The scores form each phenotype have been reversed so that a greater score indicates a greater SES. The similarity of these two distributions indicates that the UK Biobank data set is comparable to the rest of the UK. The panel on the right shows the distribution of the Income scores in UK Biobank. This indicates that most of the participants were in households where the income was between £18,000 to £31,999 and £31,000 to £51,999.

Table S1. Related to Figure 1B. Genome-wide significant SNP-based association results for household income ($P < 5 \times 10^{-8}$). The results are ordered by significance of the association. The independent SNP signals, as determined by the LD Clumping analysis, are highlighted in red. The sample size was 96,900 for each SNP presented below. Functional annotation of the household income associated genome-wide significant SNPs. All information contained in this table was extracted from the GTEx database (http://www.broadinstitute.org/gtex/) and the Regulome DB database (http://regulome.stanford.edu/index).

**SNP number**	**Chr**	**Position**	**Allele 1**	**Allele 2**	***P*-value**	**Beta**	**MAF**	**INFO**	**cis-eQTL**	**Regulome DB Score**	**Position weight matrix**	**Transcription factor binding site**	**Histone modifications**	**DNase hyper sensitive sites**	**FAIRE sites**	**DMR**
rs187848990	2	101207261	T	C	2.23×10^{-8}	0.077	0.030	0.945	no	6	yes	no	yes	no	no	no
rs7252896	19	32820876	A	T	3.11×10^{-8}	0.030	0.247	0.924	no	6	yes	no	yes	no	no	no
rs8100891	19	32829513	G	C	3.42×10^{-8}	0.029	0.261	0.998	yes	5	yes	yes	yes	no	no	no
rs7255223	19	32824310	A	C	4.27×10^{-8}	0.028	0.263	0.995	yes	4	no	yes	yes	yes	no	no

Table S3. Related to Figure 3 and Figure 4. Genetic correlations between SES as measured by social deprivation and household income from UK Biobank and the 32 health and anthropometric variables. The heritability Z-score and the mean χ^2 indicate the level of power to detect association where a heritability Z-score of >4 and a mean χ^2 >1.02 being considered well powered [S47]. Tests that withstood FDR correction are shown in bold. FDR correction indicated statistical significance at *P* = 0.0153 for social deprivation and at *P* = 0.032 for household income.

	**Social Deprivation**					**Household Income**				
**Phenotypes**	**Genetic correlation**	**Standard error**	***P*-value**	**Heritability Z-score**	**Mean χ^2**	**Genetic correlation**	**Standard error**	***P*-value**	**Heritability Z-score**	**Mean χ^2**
**Cognitive abilities**										
Childhood intelligence	0.500	0.118	**2.30×10^{-5}**	5.942	1.076	0.668	0.102	**4.96×10^{-11}**	5.8616	1.076
Years of Education	0.548	0.054	**1.80×10^{-24}**	20.687	1.372	0.903	0.040	**4.14×10^{-115}**	20.687	1.372
VNR Biobank	0.338	0.073	**3.80×10^{-6}**	10.865	1.167	0.704	0.059	**3.94×10^{-33}**	10.481	1.167
**Longevity**										
Longevity	0.301	0.1242	**0.0154**	4.127	1.038	0.303	0.107	**0.005**	4.049	1.038
**Vascular and autoimmune disease**										
Coronary artery disease	−0.029	0.075	0.700	7.954	1.145	−0.132	0.061	**0.032**	7.890	1.145
Systolic blood pressure	−0.091	0.067	0.175	12.507	1.048	−0.071	0.056	0.204	12.172	1.048
Diastolic blood pressure	−0.175	0.065	**0.007**	11.519	1.051	−0.108	0.060	0.073	10.677	1.051
Rheumatoid arthritis	−0.065	0.073	0.377	3.691	1.064	−0.092	0.073	0.205	3.814	1.064
Smoking yes/no	−0.511	0.078	**5.87×10^{-11}**	11.358	1.104	−0.321	0.062	**2.11×10^{-7}**	11.358	1.104
**Metabolic disease/phenotypes**										
Type 2 Diabetes	−0.109	0.074	0.143	9.066	1.133	−0.002	0.069	0.972	9.001	1.133
Obesity	−0.268	0.049	**3.23×10^{-8}**	17.370	1.124	−0.263	0.044	**2.11×10^{-9}**	17.556	1.124
Childhood obesity	−0.234	0.072	**0.001**	9.261	1.033	−0.016	0.067	0.809	9.357	1.033
HOMA B	−0.072	0.091	0.430	6.605	1.053	−0.158	0.088	0.073	6.145	1.053
HOMA IR	−0.189	0.115	0.101	5.342	1.053	−0.297	0.103	**0.004**	5.252	1.053

HbA1c	−0.192	0.110	0.081	5.410	1.060	−0.208	0.076	**0.006**	5.291	1.060
High density lipoprotein cholesterol	0.175	0.056	**0.002**	5.535	1.152	0.207	0.058	**3.48 × 10^{-4}**	5.546	1.152
Low density lipoprotein cholesterol	−0.179	0.074	**0.015**	3.717	1.140	−0.231	0.072	**0.001**	3.585	1.140
Triglycerides	−0.187	0.053	**4.30 × 10^{-4}**	5.931	1.153	−0.226	0.048	**2.47 × 10^{-6}**	5.937	1.153
Fasting insulin	−0.150	0.111	0.175	5.814	1.054	−0.242	0.098	**0.014**	5.867	1.054
**Psychiatric disease**										
ADHD	−0.366	0.204	0.073	2.297	1.016	−0.209	0.147	0.156	2.420	1.016
Alzheimer's 500kb	−0.041	0.105	0.698	5.531	1.105	−0.222	0.083	**0.007**	5.365	1.105
Alzheimer's	−0.060	0.127	0.636	2.127	1.114	−0.273	0.115	**0.018**	1.917	1.114
Autism	0.009	0.079	0.913	8.759	1.058	0.071	0.069	0.302	8.485	1.058
Bipolar	−0.039	0.067	0.558	10.591	1.186	0.148	0.065	**0.024**	10.405	1.186
MDD	−0.312	0.117	**0.007**	5.474	1.078	−0.326	0.101	**0.001**	5.520	1.078
Schizophrenia	−0.215	0.045	**1.66 × 10^{-6}**	22.285	1.812	−0.104	0.039	**0.009**	22.202	1.812
Neuroticism UK Biobank	−0.159	0.054	**0.003**	9.121	1.239	−0.303	0.048	**3.92 × 10^{-10}**	9.090	1.239
Neuroticism	−0.224	0.115	0.051	4.199	1.057	−0.433	0.103	**2.85 × 10^{-5}**	4.334	1.057
Meta−Neuroticism			**9.06 × 10^{-4}**							
**Anthropometric traits**										
Height	0.124	0.034	**3.00 × 10^{-4}**	17.958	2.973	0.208	0.033	**1.51 × 10^{-10}**	17.766	2.973
Head circumference	0.027	0.113	0.810	5.492	1.041	0.239	0.096	**0.013**	5.311	1.041
BMI	−0.261	0.040	**7.83 × 10^{-11}**	18.081	1.262	−0.218	0.036	**9.62 × 10^{-10}**	18.765	1.262
Birthweight	−0.021	0.094	0.826	6.109	1.062	0.131	0.080	0.102	5.735	1.062
**Neurological measures**										
ICV	0.095	0.122	0.438	3.819	1.041	0.533	0.106	**4.79 × 10^{-7}**	3.745	1.041
Hippocampal volume	0.084	0.121	0.486	3.655	1.024	0.097	0.117	0.407	3.736	1.024

Abbreviations: HOMA B, homeostatic model assessment beta-cells; HOMA IR, homeostatic model assessment insulin resistance; HbA1c, glycated haemoglobin; ADHD, attention deficit hyperactivity disorder; MDD, major depressive disorder; BMI, body mass index; ICV, intracranial volume.

Supplemental Experimental Procedures

Study design and participants

The principal data set used in this study of socioeconomic status (SES) was taken from UK Biobank (http://www.ukbiobank.ac.uk) [S48]. UK Biobank consists of 502,655 community-dwelling participants recruited between 2006 and 2010 in the United Kingdom (target age range 40-69 years). Participants gave detailed information about their background and lifestyles, underwent cognitive and physical tests, and agreed to have their health followed longitudinally. In addition, blood, urine, and saliva samples were provided for future analyses. In the current study, genome-wide genotyping data were available on 112,151 individuals (52.53% female) aged 40-73 years (mean age = 56.9 years, SD = 7.9) after the quality control process was implemented (described below). UK Biobank received ethical approval from the Research Ethics Committee (REC) (REC reference, 11/NW/0382). This study has been completed under UK Biobank application 10279.

Phenotype measurement of SES

Two measurements of SES were used in the current study. An area based measurement, The Townsend Social deprivation Index [S49], and self-reported household income. The Townsend score is a measure of the level of social deprivation for the area in which the individual lives. Each participant was assigned a Townsend score at the time of recruitment. Data from the last national census were used to derive the score for each participant based on their postcode. Four variables contribute to a participant's Townsend score: the percentage of those aged 16 or over who are unemployed, and percentages of households who do not own a car, do not own their home, and which are overcrowded. The Townsend score is an indicator of the level of social deprivation in an area, where a greater score indicates a higher level of deprivation and a lower average SES.

Self-reported household income was collected using a 5 point scale corresponding to the total household income before tax, 1 being less than £18,000, 2 being £18,000 - £29,999, 3 being £30,000 - £51,999, 4 being £52,000 – £100,000, and 5 being greater than £100,000. Participants were removed from the analysis if they answered "do not know" (n= 4319), or "prefer not to answer" (n = 10553).

In response to a reviewer's request, we investigated the degree to which multiple individuals from the same household may have contributed to UK Biobank. It should be noted that the data pertaining to participant

co-habitation is based on address and so individuals who reside in the same army barracks, care homes, hospitals etc, would count as living together. In addition these data do not take into account any changes of address since recruitment, nor the how long participants have been living in the same address. We removed one individual per household retaining the male if available in order to more closely pair the phenotype (household income) with the genotype more likely to contain causal elements, Figure S2. This resulted in a final sample size of 88,183 (47,797 males and 40,386 females). We then repeated the GWAS on this reduced data set along with the gene-based analysis, genetic correlations, and polygenic profile scores.

Genotyping and Quality Control

The 152,729 blood samples submitted to UK Biobank were genotyped using either the UKBileve array (N = 49,979) or the UK Biobank axiom array (N = 102,750). Affymetrix performed genotyping on 33 batches of ~4,700 samples and also conducted the initial quality control procedure on the genotyping data. Details of the sample processing specific to the UK Biobank project are available at http://biobank.ctsu.ox.ac.uk/crystal/refer.cgi?id=155583, and the details of the Axiom array at http://media.affymetrix.com/support/downloads/manuals/axiom_2_assay_auto_workflow_user_guide.pdf.

Prior to release of data from UK Biobank, a stringent quality control protocol was applied, and performed at the Wellcome Trust Centre for Human Genetics (WTCHG). Further details of the quality control procedure implemented by the WTCHG can be found at http://biobank.ctsu.ox.ac.uk/crystal/refer.cgi?id=155580.

Additional quality control was performed for this study. Individuals were removed based on non-British ancestry (within those who self-identified as being British, principal component analysis was used to remove outliers, *n*=32,484), high missingness (*n*=0), relatedness (*n*=7,948), QC failure in UK Bileve (*n*=187), and gender mismatch (*n*=0). A total of 112,151 individuals remained for further analyses.

Genome-wide association analyses (GWAS) in the UK Biobank sample

The UK Biobank interim release was imputed to a reference set which combined the UK10K haplotype and 1000 Genomes Phase 3 reference panels. Full details can be found at http://biobank.ctsu.ox.ac.uk/crystal/refer.cgi?id=157020. The association results were filtered to exclude variants where minor allele frequency (<0.1%) or imputation quality (<0.1) leaving a total of ~17.3 million SNPs.

Curation of summary data from GWAS on physical and psychiatric disease

Genetic correlations and polygenic profile scores were derived using the SES variables in UK Biobank and summary statistics from 32 mostly health-related phenotypes which show phenotypic correlations with SES. Table S2 provides key references showing evidence for the phenotypic associations between measures of SES and health. Full details of the prior GWAS studies which provided summary statistics along with links to the data (where applicable) are provided in Table S2.

Statistical Analysis

Genome-wide SNP-based heritability

The total phenotypic variance explained by common SNPs was estimated using GCTA-GREML [S50, S51]. All genotyped autosomal variants were included in the GCTA-GREML analyses for both the social deprivation and the household income variables from UK Biobank.

SNP-based association analyses

Association analyses for both the social deprivation phenotype and the household income phenotype were adjusted to control for the effects of age, sex, assessment centre, genotyping batch, genotyping array, and population stratification (using 10 principal components). A total of 112,005 participants had both a Townsend score and genotype data available and a total of 96,900 genotyped individuals provided data on household income. Association analyses were conducted using SNPTEST v2.5 [S52] (software available at https://mathgen.stats.ox.ac.uk/genetics_software/snptest/snptest.html#introduction). An additive model was used by specifying the 'frequentist1' option and genotype dosage scores were used to account for imputed genotype uncertainty.

Clumping

The degree to which genome-wide significant hits were tagging independent regions of the genome was examined by using linkage disequilibrium (LD) clumping in PLINK. Here, the European panel of the 1000 genomes (phase 1, release 3) was used to model the degree of LD between markers. Index SNPs, defined as those with a *P*-value of $< 5 \times 10^{-8}$, SNPs within 500kb of the index SNP and in LD of $r^2 > 0.1$ and with a *P*-value of $< 1 \times 10^{-5}$, were used to define genomic regions.

Gene-based association analysis

Gene-based association analyses were conducted using MAGMA [S53]. Summary data from the SNP-based analyses were used to derive gene-based statistics. SNPs were allocated to genes based on their position according to the NCBI 37.3 build with gene boundaries being defined as the start and stop site. The European panel of the 1000 genomes (phase 1, release 3) was used to model linkage disequilibrium. This resulted in a total of 18,061 genes being included in the gene-based analysis for social deprivation and for household income. A Bonferroni correction was used to control for multiple testing, giving an alpha level for social deprivation and for household income of 2.768×10^{-6}.

Functional annotation and gene expression

For the four genome-wide significant SNPs associated with household income (reported below), evidence of expression quantitative trait loci (eQTL) and functional annotation were explored using publicly-available online resources. The Genotype-Tissue Expression Portal (GTEx) (http://www.gtexportal.org) was used to identify eQTLs associated with the SNPs. Regulome DB [S54]. (http://www.regulomedb.org/) was used to identify regulatory DNA elements in non-coding and intergenic regions of the genome in normal cell lines and tissues.

LDS regression partitioned heritability method

Partitioned heritability can be achieved using the LDS regression method [S55]. This analysis examines groups of SNPs that share the same functional properties and is used to derive a heritability metric for each grouping. The goal of these analyses is to determine if specific groups of SNPs make a greater contribution to the total heritability estimate than would be expected by their size. This is achieved by performing multiple regression of GWAS test statistics onto LD scores for partitioned regions of the genome. This enables the percentage of a trait's total SNP-based heritability for specific regions of the genome to be derived. In order to show that a region of the genome is making a greater contribution to a phenotype than would be expected, an enrichment statistic is derived. Here enrichment is defined as the proportion of the heritability of the region, divided by the proportion of SNPs contained within it or $Pr(h^2)/Pr(SNPs)$. Should $Pr(h^2)/Pr(SNPs) = 1$, no enrichment is found, as the proportion of SNPs a region contains is equal to the heritability it tags. In instances

where $Pr(h^2)/Pr(SNPs) > 1$ the region shows evidence that it is making a greater contribution towards the heritability estimate than its size alone would suggest.

Enrichment of partitioned regions was performed separately for social deprivation and household income. Firstly a baseline model was derived using 52 overlapping, functional categories (described below). Secondly, a cell-specific model was constructed by adding each of the 10 cell-specific functional groups to the baseline model one at a time.

Multiple testing was controlled for by applying an FDR correction to the to the baseline model using 52 categories. For the cell-specific analysis the baseline model was first included and the level of enrichment for each cell specific category as derived. Here, 10 tests were controlled for using FDR.

A total of 52 overlapping categories were included in the baseline model. These were coding regions, 3'UTR, 5'UTR, promoter and intronic regions [S56, S57]. The gene sets of digital genomic footprint and transcription factor binding site were used [S57, S58]. The CTCF, promoter-flanking, transcribed, transcription start sites (TSS), strong enhancer and weak enhancer categories are included [S59]. DNase I hypersensitivity sites (DHS) were formed by utilising the data from ENCODE and from the Roadmap Epigenomes data [S60]. These were used to create two functional groups, one corresponding to all cell types and the second only those that were found within the foetal cell type. Cell type specific H3K4me1, H3K4me3, and H3K9ac data were taken from work performed on the Epigenomics Roadmap [S34]. An additional version of H3k27ac was also included [S61]. For each of these groups all cell types were used in the baseline model. Super enhancers are clusters of enhancers that show a high level of activity [S61]. This group correspond to a subset of the H3K27 annotation. Also included is a group that has been shown to be conserved along the mammalian line [S62, S63]. Finally, a group of enhancers were included that show a bidirectional capped transcript. These were identified using cap analysis of the gene expression levels in the sample panel of FANTOM5 [S64]. In order to control for SNPs within these categories tagging variance coming from outside the groupings a 500 kb boundary was included around each category and a 100bp window around the ChIP-seq peaks (regions that were DNase hypersensitive or associated with the H3K4me1, H3K4me, or the H3K9ac groupings).

The SNPs in the baseline histone mark groupings were formed by combining across tissue types. In order to determine if specific tissue types make greater contributions to SES we grouped each mark into cell

types of central nervous system, kidney, liver, cardiovascular, connective/bone, gastrointestinal, immune/hematopoietic, adrenal/pancreas, skeletal muscle, and other.

Genetic overlap with other traits

Genetic correlations can be derived by exploiting the pattern of LD found across the genome. This is due to the level of association a SNP shows in a GWAS is a product of both its own contribution toward a phenotype as well as variants that it is in LD with [S65]. In addition, SNPs in regions of high LD provide a measure of a greater proportion of the genome than SNPs in regions of low LD.

Assuming a polygenic architecture, SNPs in regions of high LD will show greater association statistics than SNPs found in regions of low LD. This means that the level of LD can be used to predict GWAS association test statistics [S47, S66]. By extending this logic to a bivariate design, the product of test statistics from each locus can be predicted using LD in the presence of a non-zero genetic correlation between pairs of traits.

Here, we use LDS regression to derive genetic correlations between SES, as measured by the Townsend Social Deprivation Index [S49] and household income, and health traits using 32 large GWAS consortia data sets to quantify the level of overlap between the genetic architecture of health traits and SES in UK Biobank. This method has been used before to establish a shared genetic component between cognitive functions and health traits [S47, S67, S68]. With regard to the analyses using the summary data from the Alzheimer's disease GWAS, due to the large effects in the *APOE* region, a 500kb region was removed from around each side of this region and the analysis was repeated. The Alzheimer's data set without this region is referred to as Alzheimer's 500kb in the Tables S3. Due to the high genetic correlation between the two measures of neuroticism used ($r_g = 1$), the *P* values derived from the genetic correlations between these two variables with social deprivation were meta-analysed using Stouffer's weighted Z [S69, S70]. This meta-analysed *P* value was then used to test whether statistical significance remained following FDR control for the tests performed.

We use the data processing pipeline described by Bulik-Sullivan et al., (2015) [S47] to derive genetic correlations between pairs of traits. A MAF of > 0.01 was used as a cut off and only those SNPs found in the HapMap3 with 1000 Genomes EUR with a MAF > 0.05 were included. The integrated_phase1_v3.20101123 was used for LDS regression. Next, indels and structural variants were removed, as were strand-ambiguous SNPs. Genome-wide significant SNPs were also removed, along with SNPs with very large effect sizes ($\chi^2 > 80$)

as the presence of outliers can increase the standard error in a regression model. LD scores and weights for use with the GWAS of European ancestry were downloaded from the Broad Institute (http://www.broadinstitute.org/~bulik/eur_ldscores/). An unconstrained intercept was used in the regression model as it was not possible to quantify the degree of sample overlap between the traits used here.

Polygenic prediction

The .map and .ped files supplied by UK Biobank were recoded to the ACGT format (from the 1, 2 numerical allele code) using a bespoke program developed by one of the authors (DCL). This program used the look-up substitution method where by a look up string hash table was created to hold the SNP-ID in addition to the allele identifiers for the SNP. A loop was conducted on the string position which created an additional string with the correct ACGT encode. This was then included to the six mandatory fields extracted from the initial string.

Polygenic profile scores were created for 28 health-related phenotypes from published GWAS in all participants with genome-wide SNP data using PRSice [S71]. Strand-ambiguous SNPs and SNPs with a minor allele frequency < 0.01 were removed prior to creating the polygenic profile scores. SNPs in linkage equilibrium with an $r^2 < 0.25$ within a 200bp window were obtained using clumping. The polygenic profile scores were then calculated by the sum of the alleles associated with the phenotype of interest across many genetic loci, weighted by their effect size estimated from the GWAS summary statistics. Five polygenic profile scores were created including variants according to the significance of their association with their phenotype, at *P*-value thresholds of 0.01, 0.05, 0.1, 0.5 and all SNPs.

The associations between the 28 polygenic profile scores and SES were examined using regression models, adjusting for age at measurement, sex, genotyping batch, genotyping array, assessment centre, and the first ten genetic principal components to adjust for population stratification. All analyses were performed in R, and all obtained *P*-values were corrected for multiple testing using the False Discovery Rate (FDR) method [S72].

A number of the data sets used in the analysis incorporating LDS regression were unsuitable for use with the polygenic profile score method and so the phenotypes of, HDL, LDL, and triglycerides had to be omitted from polygenic score analysis. In addition, the polygenic profile score method cannot be used in situations where there is sample overlap. Because of this, phenotypes within UK Biobank could not be compared with each other using this method. Sample overlap may have occurred between the GWAS consortia

used to establish genetic correlations between SES and health variables as participants from the UK were used. However, there is currently no method to quantify the degree to which this may have occurred, and therefore some of the polygenic profile scores results should be interpreted with caution. The genetic correlations are, however, robust to sample overlap [S47].

Replication

The genome wide significant SNPs from the GWAS on household income in UK Biobank were tested for replication into another measure of SES, years of education using an independent sample of ~200,000 individuals [S3]. Years of education was measured as the number of years of schooling completed. The data provided by The Social Science Genetic Association Consortium [S3] did not contain data from 23andMe or from UK Biobank, or any of the UK based cohorts. rs7252896 was not included in the years of education data and no SNPs were found with an r^2 of greater than 0.5 that were not amongst the genome wide significant SNPs from UK Biobank.

In order to further examine the degree to which the genetic architecture of social deprivation and household income as measured in the UK Biobank data set overlapped with that of years of education assembled by The Social Science Genetics Association Consortium we used Linkage Disequilibrium Score regression to derive genetic correlations. The same data processing pipeline was used as described above.

Next, using PRSice [S71], we derived polygenic profile scores using the summary statistics from the social deprivation and household income GWASs in UK Biobank and used these scores to predict social deprivation and household income in Generation Scotland: the Scottish Family Health Study (GS:SFHS) data set [S73, S74, S75]. Social deprivation was measured in GS:SFHS using the Scottish Index of Multiple Deprivation 2009 (SIMD, http://www.scotland.gov.uk/topics/statistics/simd/). In brief this measure takes small areas of Scotland which are then ranked according to seven categories each indicating SES. These are income, employment, health, education, geographic access, crime, and housing. The scores derived using the SIMD are ranked the most deprived, 1, to areas that are the least deprived 6505. Household income in GS:SFHS was measured by multiple choice where the possible answers were 1 less than £10,000, 2 between £10,000 and £30,000, 3 between £30,000 and £50,000, 4 between £50,000 and £70,000, 5 more than £70,000, and 6 prefer not to answer [S74, S75]. Individuals who responded with 6 “prefer not to answer” were excluded from the analysis. Individuals were removed if they had contributed to both GS:SFHS and UK Biobank (N = 174). Linear regression models were used to examine the associations between the polygenic profiles for the UK Biobank

household income and Townsend and the target phenotypes in GS:SFHS, adjusted for age at measurement, sex and the first five genetic principal components for population stratification. All models were corrected for multiple testing across all polygenic profile scores at all five thresholds in each cohort using the False Discovery Rate method [S76].

Using marker weights from the social deprivation GWAS in UK Biobank, highly significant associations at each *P*-value threshold with SIMD in GS:SFHS where found. The most predictive score being that which was derived using the all SNPs, (*P*-value threshold = 0.01, Beta = 0.033, SE = 0.008, r^2 = 0.001, P = 2.26 × 10^{-5}, *P*-value threshold = 0.05, Beta = 0.065, SE = 0.008, r^2 = 0.005, P = 2.26 × 10^{-5}, *P*-value threshold = 0.1, Beta = 0.072, SE = 0.008, r^2 = 0.007, P = 2.26 × 10^{-5}, *P*-value threshold = 0.5, Beta = 0.077, SE = 0.008, r^2 = 0.008, P = 2.26 × 10^{-5}, *P*-value threshold = 1, Beta = 0.079, SE = 0.008, r^2 = 0.008, P = 2.26 × 10^{-5}).

Polygenic scores derived using household income in UK Biobank predicted a significant proportion of phenotypic variance for household income in GS: SFHS at each of the *P*-value thresholds used. The most predictive polygenic score was derived using a *P*-value threshold of 0.5 (*P*-value threshold = 0.01, Beta = 0.035, SE = 0.008, r^2 = 0.001, P = 1.47 × 10^{-5}, *P*-value threshold = 0.05, Beta = 0.042, SE = 0.008, r^2 = 0.002, P = 1.82 × 10^{-7}, *P*-value threshold = 0.1, Beta = 0.046, SE = 0.008, r^2 = 0.002, P = 6.77 × 10^{-9}, *P*-value threshold = 0.5, Beta = 0.052, SE = 0.008, r^2 = 0.003, P = 5.07 × 10^{-11}, *P*-value threshold = 1, Beta = 0.051, SE = 0.008, r^2 = 0.003, P = 1.20 × 10^{-10}).

Function of genes identified by clumping and MAGMA

AF4/FMR2 Family, Member 3 (*AFF3*) encodes a tissue-restricted nuclear transcriptional activator that is preferentially expressed in lymphoid tissue, previously associated with lymphoblastic leukaemia [S77], intellectual disability [S78], and rheumatoid arthritis [S79].

Carbohydrate Sulfotransferase 10 (*CHST10*) encodes a protein necessary for synthesising the neuronally expressed carbohydrate HNK1, which is involved in neurodevelopment and synaptic plasticity [S80].

LON Peptidase N-Terminal Domain And Ring Finger 2 (*LONRF2*) has been associate with coeliac disease [S81].

Neuromedin S (*NMS*) plays an important role in regulating circadian rhythms [S82].

Phosducin-Like 3 (PDCL3) is involved in the process of angiogenesis [S83].

KAT8 Regulatory NSL Complex Subunit 1 (*KANSL1*) is involved in chromatin modification and has previously been associated with intellectual disability [S84].

Macrophage Stimulating 1 (*MST1*) belongs to a family of kinases that are associated with a number of pathologies, including cancer, endothelial malformations and autoimmune disease [S85].

Expression levels of Ring Finger Protein 123 (RNF123) are associated with depression [S86].

Mutations in Microtubule-Associated Protein Tau (*MAPT*) have been associated with several neurodegenerative diseases, including Alzheimer's disease [S87].

Acylaminoacyl-Peptide Hydrolase (APEH) has an antioxidant function and has been associated with various cancers [S88].

Bassoon Presynaptic Cytomatrix Protein (*BSN*) encodes a scaffold protein expressed in the brain, is involved with neurotransmitter release and was previously associated with Crohn's disease [S89] and more recently with self-rated health [S90].

Pleckstrin Homology Domain Containing, Family M (With RUN Domain) Member 1 (*PLEKHM1*) encodes a protein essential for bone resorption and variants within the gene are associated with osteopetrosis [S91].

Sarcoglycan, Delta (35kDa Dystrophin-Associated Glycoprotein) (*SGCD*) encodes a subcomplex of the dystrophin-glycoprotein complex. Mutations in this gene have been associated with limb-girdle muscular dystrophy type 2F [S92] and cardiomyopathy [S93].

Dystroglycan 1 (Dystrophin-Associated Glycoprotein 1) (*DAG1*) encodes a laminin binding component of the dystrophin-glycoprotein complex. Mutations in *DAG1* are associated with a number of muscular dystrophies [S94].

Genetic variants in the Corticotropin Releasing Hormone Receptor 1 (*CRHR1*) have been associated with alcoholism [S95] and anxiety disorders [S96].

Aminomethyltransferase (*AMT*) encodes one of four critical components of the glycine cleavage system. Mutations in this gene have been associated with glycine encephalopathy [S97].

Zinc Finger, DHHC-Type Containing 11 (*ZDHHC11*) is located in a genomic region associated with lung [S98] and bladder cancers [S99].

1-Aminocyclopropane-1-Carboxylate Synthase Homolog (Arabidopsis)(Non-Functional)-Like (*ACCSL*)

KANSL1 MAPT, *PLEKHM1* and *CRHR1* are in a region on chromosome 17 recently associated with Alzheimer's Disease [S100].

Comparison between income in the full data set and income in the restricted sample of 88,183

We first performed GCTA-GREML using the reduced sample size and found a very similar heritability estimate of 12% (SE = 0.7%). The results of the GWAS can be seen in Figure S3, shows the genome wide significant SNPs. The results of the GWAS on the reduced data set revealed 12 new genome-wide significant SNPs in 2 independent regions on chromosome 9. The SNP rs139128645, also on chromosome 9, did fall within an intron in the *EHMT1* gene. This gene has previously been associated with Kleefstra syndrome [S101] which includes symptoms such as developmental delay as well as learning difficulties. Another result of the loss of sample size was that the significant SNPs found using the whole sample were no longer significant at the genome-wide threshold of 5×10^{-8}. The results indicate that the beta weights and the standard errors of the genome-wide significant SNPs for both the reduced and full data sets were highly similar and that the fluctuation of sample size between these two comparisons is the most likely reason for the small difference in the *P*-values found between the full and reduced sample size, rather than any bias introduced from individuals residing together. This conclusion is also supported by the results of the gene based statistics, genetic correlations, partitioned heritability and the polygenic profile scores (as the genetic overlap between income and the health and anthropometric traits is very similar, along with the proportion of variance explained using the polygenic profile scores. These follow up results are available from the author. Additionally, as for the full sample, enrichment was found for SNPs in within 500bp of H3K9ac SNPs as well as those found in conserved regions, but not for SNPs within 500 bp of the DHS and conserved regions.

Genetic Consortia Acknowledgements

Social Science Genetics Association Consortium

This research was facilitated by the Social Science Genetic Association Consortium (SSGAC)

International Consortium for Blood Pressure

Blood pressure data was provided by the Association Consortium (SSGAC), and the International Consortium for Blood Pressure (ICBP)

CHARGE-Aging and Longevity

Longevity data have been provided by the CHARGE-Aging and Longevity consortium. Longevity was defined as reaching age 90 years or older. Genotyped participants who died between the ages of 55 and 80 years were used as the control group. There were 6036 participants who achieved longevity and 3757 participants in the control group across participating studies in the discovery meta-analysis.

Broer L, Buchman AS, Deelen J, Evans DS, Faul JD, Lunetta KL, Sebastiani P, Smith JA, Smith AV, Tanaka T, Yu L, Arnold AM, Aspelund T, Benjamin EJ, De Jager PL, Eirkisdottir G, Evans DA, Garcia ME, Hofman A, Kaplan RC, Kardia SL, Kiel DP, Oostra BA, Orwoll ES, Parimi N, Psaty BM, Rivadeneira F, Rotter JI, Seshadri S, Singleton A, Tiemeier H, Uitterlinden AG, Zhao W, Bandinelli S, Bennett DA, Ferrucci L, Gudnason V, Harris TB, Karasik D, Launer LJ, Perls TT, Slagboom PE, Tranah GJ, Weir DR, Newman AB, van Duijn CM and Murabito JM. **GWAS of Longevity in CHARGE Consortium Confirms APOE and FOXO3 Candidacy**. *J Gerontol A Biol Sci Med Sci*. 2015;70:110-8.

Acknowledgments

The CHARGE Aging and Longevity working group analysis of the longevity phenotype was funded through the individual contributing studies. The working group thanks all study participants and study staff**.**

International Genomics of Alzheimer’s Project (IGAP)

Alzheimer’s disease data were obtained from (IGAP)

Material and methods

International Genomics of Alzheimer's Project (IGAP) is a large two-stage study based upon genome-wide association studies (GWAS) on individuals of European ancestry. In stage 1, IGAP used genotyped and imputed data on 7 055 881 single nucleotide polymorphisms (SNPs) to meta-analyse four previously-published GWAS datasets consisting of 17 008 Alzheimer's disease cases and 37 154 controls (The European Alzheimer's disease Initiative – EADI the Alzheimer Disease Genetics Consortium – ADGC The Cohorts for Heart and Aging Research in Genomic Epidemiology consortium – CHARGE The Genetic and Environmental Risk in AD consortium – GERAD). In stage 2, 11 632 SNPs were genotyped and tested for association in an independent set of 8572 Alzheimer's disease cases and 11 312 controls. Finally, a meta-analysis was performed combining results from stages 1 & 2. Only stage 1 data were used for LD Score regression.

Acknowledgments

We thank the International Genomics of Alzheimer's Project (IGAP) for providing summary results data for these analyses. The investigators within IGAP contributed to the design and implementation of IGAP and/or provided data but did not participate in analysis or writing of this report. IGAP was made possible by the generous participation of the control subjects, the patients, and their families. The i–Select chips was funded by the French National Foundation on Alzheimer's disease and related disorders. EADI was supported by the LABEX (laboratory of excellence program investment for the future) DISTALZ grant, Inserm, Institut Pasteur de Lille, Université de Lille 2 and the Lille University Hospital. GERAD was supported by the Medical Research Council (Grant n° 503480), Alzheimer's Research UK (Grant n° 503176), the Wellcome Trust (Grant n° 082604/2/07/Z) and German Federal Ministry of Education and Research (BMBF): Competence Network Dementia (CND) grant n° 01GI0102, 01GI0711, 01GI0420. CHARGE was partly supported by the NIH/NIA grant R01 AG033193 and the NIA AG081220 and AGES contract N01–AG–12100, the NHLBI grant R01 HL105756, the Icelandic Heart Association, and the Erasmus Medical Center and Erasmus University. ADGC was supported by the NIH/NIA grants: U01 AG032984, U24 AG021886, U01 AG016976, and the Alzheimer's Association grant ADGC–10–196728.

Supplemental References

S1. Strenze, T. (2007). Intelligence and socioeconomic success: A meta-analytic review of longitudinal research. Intelligence *35*, 401-426.

S2. Deary, I.J., Taylor, M.D., Hart, C.L., Wilson, V., Smith, G.D., Blane, D., and Starr, J.M. (2005). Intergenerational social mobility and mid-life status attainment: Influences of childhood intelligence, childhood social factors, and education. Intelligence *33*, 455-472.

S3. Okbay, A., Beauchamp, J.P., Fontana, M.A., Lee, J.J., Pers, T.H., Rietveld, C.A., Turley, P., Chen, G.-B., Emilsson, V., Meddens, S.F.W., et al. (2016). Genome-wide association study identifies 74 loci associated with educational attainment. Nature *533*, 539-542.

S4. Von Stumm, S., and Plomin, R. (2015). Socioeconomic status and the growth of intelligence from infancy through adolescence. Intelligence *48*, 30-36.

S5. Benyamin, B., Pourcain, B., Davis, O.S., Davies, G., Hansell, N.K., Brion, M.J., Kirkpatrick, R.M., Cents, R.A., Franic, S., Miller, M.B., et al. (2014). Childhood intelligence is heritable, highly polygenic and associated with FNBP1L. Mol. Psychiatry *19*, 253–258.

S6. Batty, G.D., Der, G., Macintyre, S., and Deary, I.J. (2006). Does IQ explain socioeconomic inequalities in health? Evidence from a population based cohort study in the west of Scotland. Bmj *332*, 580-584.

S7. Broer, L., Buchman, A.S., Deelen, J., Evans, D.S., Faul, J.D., Lunetta, K.L., Sebastiani, P., Smith, J.A., Smith, A.V., Tanaka, T., et al. (2015). GWAS of longevity in CHARGE consortium confirms APOE and FOXO3 candidacy. The Journals of Gerontology Series A: Biological Sciences and Medical Sciences *70*, 110-118.

S8. Schunkert, H., König, I.R., Kathiresan, S., Reilly, M.P., Assimes, T.L., Holm, H., Preuss, M., Stewart, A.F., Barbalic, M., Gieger, C., et al. (2011). Large-scale association analysis identifies 13 new susceptibility loci for coronary artery disease. Nat. Genet. *43*, 333-338.

S9. Colhoun, H.M., Hemingway, H., and Poulter, N.R. (1998). Socio-economic status and blood pressure: an overview analysis. Journal of human hypertension *12*, 91-110.

S10. International Consortium for Blood Pressure Genome-Wide Association Studies (2011). Genetic variants in novel pathways influence blood pressure and cardiovascular disease risk. Nature *478*, 103-109.

S11. Bengtsson, C., Nordmark, B., Klareskog, L., Lundberg, I., and Alfredsson, L. (2005). Socioeconomic status and the risk of developing rheumatoid arthritis: results from the Swedish EIRA study. Annals of the rheumatic diseases *64*, 1588-1594.

S12. Stahl, E.A., Raychaudhuri, S., Remmers, E.F., Xie, G., Eyre, S., Thomson, B.P., Li, Y., Kurreeman, F.A., Zhernakova, A., Hinks, A., et al. (2010). Genome-wide association study meta-analysis identifies seven new rheumatoid arthritis risk loci. Nat. Genet. *42*, 508-514.

S13. Laaksonen, M., Rahkonen, O., Karvonen, S., and Lahelma, E. (2005). Socioeconomic status and smoking. The European Journal of Public Health *15*, 262-269.

S14. Tobacco and Genetics Consortium (2010). Genome-wide meta-analyses identify multiple loci associated with smoking behavior. Nat. Genet. *42*, 441-447.

S15. Connolly, V., Unwin, N., Sherriff, P., Bilous, R., and Kelly, W. (2000). Diabetes prevalence and socioeconomic status: a population based study showing increased prevalence of type 2 diabetes mellitus in deprived areas. Journal of Epidemiology and Community Health *54*, 173-177.

S16. McLaren, L. (2007). Socioeconomic status and obesity. Epidemiologic reviews *29*, 29-48.

S17. Berndt, S.I., Gustafsson, S., Mägi, R., Ganna, A., Wheeler, E., Feitosa, M.F., Justice, A.E., Monda, K.L., Croteau-Chonka, D.C., Day, F.R., et al. (2013). Genome-wide meta-analysis

identifies 11 new loci for anthropometric traits and provides insights into genetic architecture. Nat. Genet. *45*, 501-512.
S18. Stamatakis, E., Wardle, J., and Cole, T.J. (2010). Childhood obesity and overweight prevalence trends in England: evidence for growing socioeconomic disparities. International journal of obesity *34*, 41-47.
S19. Early Growth Genetics (EGG) Consortium (2012). A genome-wide association meta-analysis identifies new childhood obesity loci. Nat. Genet. *44*, 526-531.
S20. Lehman, B.J., Taylor, S.E., Kiefe, C.I., and Seeman, T.E. (2005). Relation of childhood socioeconomic status and family environment to adult metabolic functioning in the CARDIA study. Psychosomatic medicine *67*, 846-854.
S21. Dupuis, J., Langenberg, C., Prokopenko, I., Saxena, R., Soranzo, N., Jackson, A.U., Wheeler, E., Glazer, N.L., Bouatia-Naji, N., Gloyn, A.L., et al. (2010). New genetic loci implicated in fasting glucose homeostasis and their impact on type 2 diabetes risk. Nat. Genet. *42*, 105-116.
S22. Soranzo, N., Sanna, S., Wheeler, E., Gieger, C., Radke, D., Dupuis, J., Bouatia-Naji, N., Langenberg, C., Prokopenko, I., Stolerman, E., et al. (2010). Common variants at 10 genomic loci influence hemoglobin A1C levels via glycemic and nonglycemic pathways. Diabetes *59*, 3229-3239.
S23. Teslovich, T.M., Musunuru, K., Smith, A.V., Edmondson, A.C., Stylianou, I.M., Koseki, M., Pirruccello, J.P., Ripatti, S., Chasman, D.I., Willer, C.J., et al. (2010). Biological, clinical and population relevance of 95 loci for blood lipids. Nature *466*, 707-713.
S24. Russell, A.E., Ford, T., Williams, R., and Russell, G. (2015). The Association Between Socioeconomic Disadvantage and Attention Deficit/Hyperactivity Disorder (ADHD): A Systematic Review. Child Psychiatry & Human Development, 1-19.
S25. Evans, D.A., Hebert, L.E., Beckett, L.A., Scherr, P.A., Albert, M.S., Chown, M.J., Pilgrim, D.M., and Taylor, J.O. (1997). Education and other measures of socioeconomic status and risk of incident Alzheimer disease in a defined population of older persons. Arch. Neurol. *54*, 1399-1405.
S26. Lambert, J.-C., Ibrahim-Verbaas, C.A., Harold, D., Naj, A.C., Sims, R., Bellenguez, C., Jun, G., DeStefano, A.L., Bis, J.C., Beecham, G.W., et al. (2013). Meta-analysis of 74,046 individuals identifies 11 new susceptibility loci for Alzheimer's disease. Nat. Genet. *45*, 1452-1458.
S27. Idring, S., Magnusson, C., Lundberg, M., Ek, M., Rai, D., Svensson, A.C., Dalman, C., Karlsson, H., and Lee, B.K. (2014). Parental age and the risk of autism spectrum disorders: findings from a Swedish population-based cohort. Int. J. Epidemiol., dyt262.
S28. Cross-Disorder Group of the Psychiatric Genomics Consortium (2013). Identification of risk loci with shared effects on five major psychiatric disorders: a genome-wide analysis. Lancet *381*, 1371–1379.
S29. Schoeyen, H.K., Birkenaes, A.B., Vaaler, A.E., Auestad, B.H., Malt, U.F., Andreassen, O.A., and Morken, G. (2011). Bipolar disorder patients have similar levels of education but lower socio-economic status than the general population. Journal of affective disorders *129*, 68-74.
S30. Sklar, P., Ripke, S., Scott, L.J., Andreassen, O.A., Cichon, S., Craddock, N., Edenberg, H.J., Nurnberger, J.I., Rietschel, M., and Blackwood, D. (2011). Large-scale genome-wide association analysis of bipolar disorder identifies a new susceptibility locus near ODZ4. Nat. Genet. *43*, 977-983.
S31. Muntaner, C., Eaton, W.W., Diala, C., Kessler, R.C., and Sorlie, P.D. (1998). Social class, assets, organizational control and the prevalence of common groups of psychiatric disorders. Social science & medicine *47*, 2043-2053.
S32. Ripke, S., Wray, N.R., Lewis, C.M., Hamilton, S.P., Weissman, M.M., Breen, G., Byrne, E.M., Blackwood, D.H., Boomsma, D.I., Cichon, S., et al. (2013). A mega-analysis of genome-wide association studies for major depressive disorder. Mol. Psychiatry *18*, 497-511.
S33. Dohrenwend, B.P. (1990). Socioeconomic status (SES) and psychiatric disorders. Social psychiatry and psychiatric epidemiology *25*, 41-47.

S34. Schizophrenia Working Group of the Psychiatric Genomics Consortium (2014). Biological insights from 108 schizophrenia-associated genetic loci. Nature *511*, 421-427.

S35. Jonassaint, C.R., Siegler, I.C., Barefoot, J.C., Edwards, C.L., and Williams, R.B. (2011). Low life course socioeconomic status (SES) is associated with negative NEO PI-R personality patterns. International journal of behavioral medicine *18*, 13-21.

S36. De Moor, M.H., Van Den Berg, S.M., Verweij, K.J., Krueger, R.F., Luciano, M., Vasquez, A.A., Matteson, L.K., Derringer, J., Esko, T., Amin, N., et al. (2015). Meta-analysis of genome-wide association studies for neuroticism, and the polygenic association with major depressive disorder. JAMA psychiatry *72*, 642-650.

S37. Huang, Y., van Poppel, F., and Lumey, L.H. (2015). Differences in height by education among 371,105 Dutch military conscripts. Economics & Human Biology *17*.

S38. Wood, A.R., Esko, T., Yang, J., Vedantam, S., Pers, T.H., Gustafsson, S., Chu, A.Y., Estrada, K., Luan, J.a., Kutalik, Z., et al. (2014). Defining the role of common variation in the genomic and biological architecture of adult human height. Nat. Genet. *46*, 1173-1186.

S39. Fotenos, A.F., Mintun, M.A., Snyder, A.Z., Morris, J.C., and Buckner, R.L. (2008). Brain volume decline in aging: evidence for a relation between socioeconomic status, preclinical Alzheimer disease, and reserve. Archives of Neurology *65*, 113-120.

S40. Taal, H.R., St Pourcain, B., Thiering, E., Das, S., Mook-Kanamori, D.O., Warrington, N.M., Kaakinen, M., Kreiner-Møller, E., Bradfield, J.P., Freathy, R.M., et al. (2012). Common variants at 12q15 and 12q24 are associated with infant head circumference. Nat. Genet. *44*, 532-538.

S41. Molarius, A., Seidell, J.C., Sans, S., Tuomilehto, J., and Kuulasmaa, K. (2000). Educational level, relative body weight, and changes in their association over 10 years: an international perspective from the WHO MONICA Project. American Journal of Public Health *90*, 1260-1268.

S42. Locke, A.E., Kahali, B., Berndt, S.I., Justice, A.E., Pers, T.H., Day, F.R., Powell, C., Vedantam, S., Buchkovich, M.L., Yang, J., et al. (2015). Genetic studies of body mass index yield new insights for obesity biology. Nature *518*, 197-206.

S43. Spencer, N., Bambang, S., Logan, S., and Gill, L. (1999). Socioeconomic status and birth weight: comparison of an area-based measure with the Registrar General's social class. Journal of Epidemiology and Community Health *53*, 495-498.

S44. Horikoshi, M., Yaghootkar, H., Mook-Kanamori, D.O., Sovio, U., Taal, H.R., Hennig, B.J., Bradfield, J.P., St Pourcain, B., Evans, D.M., Charoen, P., et al. (2013). New loci associated with birth weight identify genetic links between intrauterine growth and adult height and metabolism. Nat. Genet. *45*, 76-82.

S45. Hibar, D.P., Stein, J.L., Renteria, M.E., Arias-Vasquez, A., Desrivières, S., Jahanshad, N., Toro, R., Wittfeld, K., Abramovic, L., Andersson, M., et al. (2015). Common genetic variants influence human subcortical brain structures. Nature *520*, 224-229.

S46. Rao, H., Betancourt, L., Giannetta, J.M., Brodsky, N.L., Korczykowski, M., Avants, B.B., Gee, J.C., Wang, J., Hurt, H., Detre, J.A., et al. (2010). Early parental care is important for hippocampal maturation: evidence from brain morphology in humans. NeuroImage *49*, 1144-1150.

S47. Bulik-Sullivan, B., Finucane, H.K., Anttila, V., Gusev, A., Day, F.R., Loh, P.-R., Duncan, L., Perry, J.R., Patterson, N., Robinson, E.B., et al. (2015). An atlas of genetic correlations across human diseases and traits. Nat. Genet.

S48. Sudlow, C., Gallacher, J., Allen, N., Beral, V., Burton, P., Danesh, J., Downey, P., Elliott, P., Green, J., Landray, M., et al. (2015). UK biobank: an open access resource for identifying the causes of a wide range of complex diseases of middle and old age. PLoS Med *12*, e1001779.

S49. Townsend, P. (1987). Deprivation. Journal of social policy. 16 *2*.

S50. Yang, J., Lee, S.H., Goddard, M.E., and Visscher, P.M. (2011). GCTA: a tool for genome-wide complex trait analysis. The American Journal of Human Genetics *88*, 7.

S51. Yang, J., Benyamin, B., McEvoy, B.P., Gordon, S., Henders, A.K., Nyholt, D.R., Madden, P.A., Heath, A.C., Martin, N.G., Montgomery, G.W., et al. (2010). Common SNPs explain a large proportion of the heritability for human height. Nat. Genet. *42*, 4.

S52. Marchini, J., Howie, B., Myers, S., McVean, G., and Donnelly, P. (2007). A new multipoint method for genome-wide association studies by imputation of genotypes. Nature genetics *39*, 906-913.

S53. de Leeuw, C.A., Mooij, J.M., Heskes, T., and Posthuma, D. (2015). MAGMA: Generalized Gene-Set Analysis of GWAS Data. 11 *4*.

S54. Boyle, A.P., Hong, E.L., Hariharan, M., Cheng, Y., Schaub, M.A., Kasowski, M., Karczewski, K.J., Park, J., Hitz, B.C., Weng, S., et al. (2012). Annotation of functional variation in personal genomes using RegulomeDB. Genome Res. *22*, 1790-1797.

S55. Finucane, H.K., Bulik-Sullivan, B., Gusev, A., Trynka, G., Reshef, Y., Loh, P.-R., Anttila, V., Xu, H., Zang, C., Farh, K., et al. (2015). Partitioning heritability by functional annotation using genome-wide association summary statistics. Nat. Genet. *47*, 1228-1235.

S56. Kent, W.J., Sugnet, C.W., Furey, T.S., Roskin, K.M., Pringle, T.H., Zahler, A.M., and Haussler, D. (2002). The human genome browser at UCSC. Genome research *12*, 996-1006.

S57. Gusev, A., Lee, S.H., Trynka, G., Finucane, H., Vilhjálmsson, B.J., Xu, H., Zang, C., Ripke, S., Bulik-Sullivan, B., Stahl, E., et al. (2014). Partitioning heritability of regulatory and cell-type-specific variants across 11 common diseases. The American Journal of Human Genetics *95*, 535-552.

S58. ENCODE Project Consortium. (2012). An integrated encyclopedia of DNA elements in the human genome. Nature *489*, 57-74.

S59. Hoffman, M.M., Ernst, J., Wilder, S.P., Kundaje, A., Harris, R.S., Libbrecht, M., Giardine, B., Ellenbogen, P.M., Bilmes, J.A., Birney, E., et al. (2012). Integrative annotation of chromatin elements from ENCODE data. Nucleic Acids Res., gks1284.

S60. Trynka, G., Sandor, C., Han, B., Xu, H., Stranger, B.E., Liu, X.S., and Raychaudhuri, S. (2013). Chromatin marks identify critical cell types for fine mapping complex trait variants. Nature genetics *45*, 124-130.

S61. Hnisz, D., Abraham, B.J., Lee, T.I., Lau, A., Saint-André, V., Sigova, A.A., Hoke, H.A., and Young, R.A. (2013). Super-enhancers in the control of cell identity and disease. Cell *155*, 934-947.

S62. Lindblad-Toh, K., Garber, M., Zuk, O., Lin, M.F., Parker, B.J., Washietl, S., Kheradpour, P., Ernst, J., Jordan, G., Mauceli, E., et al. (2011). A high-resolution map of human evolutionary constraint using 29 mammals. Nature *478*, 476-482.

S63. Ward, L.D., and Kellis, M. (2012). Evidence of abundant purifying selection in humans for recently acquired regulatory functions. Science *337*, 1675-1678.

S64. Andersson, R., Gebhard, C., Miguel-Escalada, I., Hoof, I., Bornholdt, J., Boyd, M., Chen, Y., Zhao, X., Schmidl, C., Suzuki, T., et al. (2014). An atlas of active enhancers across human cell types and tissues. Nature *507*, 455-461.

S65. Yang, J., Weedon, M.N., Purcell, S., Lettre, G., Estrada, K., Willer, C.J., Smith, A.V., Ingelsson, E., O'Connell, J.R., Mangino, M., et al. (2011). Genomic inflation factors under polygenic inheritance. Eur. J. Hum. Genet. *19*, 807-812.

S66. Bulik-Sullivan, B.K., Loh, P.-R., Finucane, H.K., Ripke, S., Yang, J., Patterson, N., Daly, M.J., Price, A.L., Neale, B.M., and Consortium, S.W.G.o.t.P.G. (2015). LD Score regression distinguishes confounding from polygenicity in genome-wide association studies. Nat. Genet. *47*, 291-295.

S67. Hill, W.D., Davies, G., The CHARGE Cognitive Working Group, Liewald, D.C., McIntosh, A.M., and Deary, I.J. (2015). Age-dependent pleiotropy between general cognitive function and major psychiatric disorders. Biol. Psychiatry.

S68. Krapohl, E., Euesden, J., Zabaneh, D., Pingault, J., Rimfeld, K., Von Stumm, S., Dale, P., Breen, G., O'Reilly, P., and Plomin, R. (2015). Phenome-wide analysis of genome-wide polygenic scores. Mol. Psychiatry.

S69. Whitlock, M.C. (2005). Combining probability from independent tests: the weighted Z-method is superior to Fisher's approach. J. Evol. Biol. *18*, 1368-1373.

S70. Zaykin, D.V. (2011). Optimally weighted Z-test is a powerful method for combining probabilities in meta-analysis. J. Evol. Biol. *24*, 1836-1841.

S71. Euesden, J., Lewis, C.M., and O'Reilly, P.F. (2015). PRSice: Polygenic Risk Score software. Bioinformatics *31*, 1466-1468.

S72. Benjamini, Y., and Hochberg, Y. (1995). Controlling the false discovery rate: a practical and powerful approach to multiple testing. Journal of the Royal Statistical Society Series B (Methodological) *57*, 289-300.

S73. Marioni, R.E., Davies, G., Hayward, C., Liewald, D., Kerr, S.M., Campbell, A., Luciano, M., Smith, B.H., Padmanabhan, S., Hocking, L.J., et al. (2014). Molecular genetic contributions to socioeconomic status and intelligence. Intelligence *44*, 26-32.

S74. Smith, B.H., Campbell, A., Linksted, P., Fitzpatrick, B., Jackson, C., Kerr, S.M., Deary, I.J., MacIntyre, D.J., Campbell, H., McGilchrist, M., et al. (2012). Cohort profile: Generation Scotland: Scottish Family Health Study (GS: SFHS). The study, its participants and their potential for genetic research on health and illness. Int. J. Epidemiol. *42*, 689-700.

S75. Smith, B.H., Campbell, H., Blackwood, D., Connell, J., Connor, M., Deary, I.J., Dominiczak, A.F., Fitzpatrick, B., Ford, I., Jackson, C., et al. (2006). Generation Scotland: the Scottish Family Health Study; a new resource for researching genes and heritability. BMC Medical Genetics *7*, 9.

S76. Benjamini, Y., and Hochberg, Y. (1995). Controlling the false discovery rate: a practical and powerful approach to multiple testing. Journal of the Royal Statistical Society. Series B (Methodological) *57*, 289-300.

S77. von Bergh, A.R., Beverloo, H.B., Rombout, P., van Wering, E.R., van Weel, M.H., Beverstock, G.C., Kluin, P.M., Slater, R.M., and Schuuring, E. (2002). LAF4, an AF4-related gene, is fused to MLL in infant acute lymphoblastic leukemia. Genes Chromosomes Cancer *35*, 92-96.

S78. Melko, M., Douguet, D., Bensaid, M., Zongaro, S., Verheggen, C., Gecz, J., and Bardoni, B. (2011). Functional characterization of the AFF (AF4/FMR2) family of RNA-binding proteins: insights into the molecular pathology of FRAXE intellectual disability. Human molecular genetics *20*, 1873-1885.

S79. Barton, A., Eyre, S., Ke, X., Hinks, A., Bowes, J., Flynn, E., Martin, P., Wilson, A.G., Morgan, A.W., Emery, P., et al. (2009). Identification of AF4/FMR2 family, member 3 (AFF3) as a novel rheumatoid arthritis susceptibility locus and confirmation of two further pan-autoimmune susceptibility genes. Hum. Mol. Genet. *18*, 2518-2522.

S80. Ong, E., Yeh, J.C., Ding, Y., Hindsgaul, O., and Fukuda, M. (1998). Expression cloning of a human sulfotransferase that directs the synthesis of the HNK-1 glycan on the neural cell adhesion molecule and glycolipids. Journal of Biological Chemistry *273*, 5190-5195.

S81. Trynka, G., Zhernakova, A., Romanos, J., Franke, L., Hunt, K., Turner, G., Bruinenberg, M., Heap, G., Platteel, M., Ryan, A., et al. (2009). Coeliac disease-associated risk variants in TNFAIP3 and REL implicate altered NF-κB signalling. Gut *58*, 1078-1083.

S82. Mitchell, J.D., Maguire, J.J., and Davenport, A.P. (2009). Emerging pharmacology and physiology of neuromedin U and the structurally related peptide neuromedin S. British journal of pharmacology *158*, 87-103.

S83. Srinivasan, S., Meyer, R.D., Lugo, R., and Rahimi, N. (2013). Identification of PDCL3 as a novel chaperone protein involved in the generation of functional VEGF receptor 2. Journal of Biological Chemistry *288*, 23171-23181.

S84. Zollino, M., Marangi, G., Ponzi, E., Orteschi, D., Ricciardi, S., Lattante, S., Murdolo, M., Battaglia, D., Contaldo, I., Mercuri, E., et al. (2015). Intragenic KANSL1 mutations and

chromosome 17q21. 31 deletions: broadening the clinical spectrum and genotype–phenotype correlations in a large cohort of patients. J. Med. Genet., jmedgenet-2015-103184.
S85. Thompson, B.J., and Sahai, E. (2015). MST kinases in development and disease. The Journal of cell biology *210*, 871-882.
S86. Glahn, D.C., Curran, J.E., Winkler, A.M., Carless, M.A., Kent, J.W., Charlesworth, J.C., Johnson, M.P., Göring, H.H., Cole, S.A., Dyer, T.D., et al. (2012). High dimensional endophenotype ranking in the search for major depression risk genes. Biol. Psychiatry *71*, 6-14.
S87. Zhang, C.C., Xing, A., Tan, M.S., Tan, L., and Yu, J.T. (2015). The Role of MAPT in Neurodegenerative Diseases: Genetics, Mechanisms and Therapy. Molecular Neurobiology *1-12*.
S88. Bergamo, P., Cocca, E., Palumbo, R., Gogliettino, M., Rossi, M., and Palmieri, G. (2013). RedOx Status, Proteasome and APEH: Insights into Anticancer Mechanisms of t10, c12-Conjugated Linoleic Acid Isomer on A375 Melanoma Cells. PLoS One *8*, 900–906.
S89. Burton, P.R., Clayton, D.G., Cardon, L.R., Craddock, N., Deloukas, P., Duncanson, A., and Green, E.K. (2007). Genome-wide association study of 14,000 cases of seven common diseases and 3,000 shared controls. Nature *447*, 17.
S90. Harris, S.E., Hagenaars, S.P., Davies, G., Hill, W.D., Liewald, D.C., Ritchie, S.J., Marioni, R.E., Sudlow, C.L., Wardlaw, J.M., McIntosh, A.M., et al. (2015). Molecular genetic contributions to self-rated health. bioRxiv, 029504.
S91. Van Wesenbeeck, L., Odgren, P.R., Coxon, F.P., Frattini, A., Moens, P., Perdu, B., MacKay, C.A., Van Hul, E., Timmermans, J.-P., Vanhoenacker, F., et al. (2007). Involvement of PLEKHM1 in osteoclastic vesicular transport and osteopetrosis in incisors absent rats and humans. The Journal of clinical investigation *117*, 919-930.
S92. Nigro, V., and Savarese, M. (2014). Genetic basis of limb-girdle muscular dystrophies: the 2014 update. Acta Myologica *33*, 1.
S93. Jacoby, D., and McKenna, W.J. (2011). Genetics of inherited cardiomyopathy. European heart journal.
S94. Godfrey, C., Clement, E., Mein, R., Brockington, M., Smith, J., Talim, B., Straub, V., Robb, S., Quinlivan, R., Feng, L., et al. (2007). Refining genotype–phenotype correlations in muscular dystrophies with defective glycosylation of dystroglycan. Brain *130*, 2725-2735.
S95. Enoch, M.A. (2013). Genetic influences on the development of alcoholism. Current psychiatry reports *15*, 1-9.
S96. Domschke, K., and Maron, E. (2013). Genetic factors in anxiety disorders. Mod Trends Pharmacopsychiatry *29*, 24-46.
S97. Kure, S., Kato, K., Dinopoulos, A., Gail, C., degrauw, T.J., Christodoulou, J., Bzduch, V., Kalmanchey, R., Fekete, G., Trojovsky, A., et al. (2006). Comprehensive mutation analysis of GLDC, AMT, and GCSH in nonketotic hyperglycinemia. Hum. Mutat. *27*, 343-352.
S98. Kang, J.U., Koo, S.H., Kwon, K.C., Park, J.W., and Kim, J.M. (2008). Gain at chromosomal region 5p15. 33, containing TERT, is the most frequent genetic event in early stages of non-small cell lung cancer. Cancer genetics and cytogenetics *182*, 1-11.
S99. Yamamoto, Y., Chochi, Y., Matsuyama, H., Eguchi, S., Kawauchi, S., Furuya, T., Oga, A., Kang, J.J., Naito, K., and Sasaki, K. (2007). Gain of 5p15. 33 is associated with progression of bladder cancer. Oncology *72*, 132-138.
S100. Jun, G., Ibrahim-Verbaas, C., Vronskaya, M., Lambert, J., Chung, J., Naj, A., Kunkle, B., Wang, L., Bis, J., Bellenguez, C., et al. (2015). A novel Alzheimer disease locus located near the gene encoding tau protein. Mol. Psychiatry.
S101. Kleefstra, T., Smidt, M., Banning, M., Oudakker, A., Van Esch, H., De Brouwer, A., Nillesen, W., Sistermans, E., Hamel, B., and De Bruijn, D. (2005). Disruption of the gene Euchromatin Histone Methyl Transferase1 (Eu-HMTase1) is associated with the 9q34 subtelomeric deletion syndrome. J. Med. Genet. *42*, 299-306.
